# Sex differences in major cardiovascular outcomes and fractures in patients with subclinical thyroid dysfunction: a systematic review and meta-analysis

**DOI:** 10.18632/aging.204352

**Published:** 2022-10-25

**Authors:** Hongjuan Fang, Runsheng Zhao, Shuang Cui, Weiqing Wan

**Affiliations:** 1 Department of Endocrinology, Beijing Tiantan Hospital, Capital Medical University, Beijing, China; 2 Department of Neurosurgery, Beijing Tiantan Hospital, Capital Medical University, Beijing, China; 3 National Clinical Research Center for Neurological Diseases, Center for Stroke, Beijing Institute for Brain Disorders, Beijing Key Laboratory of Translational Medicine for Cerebrovascular Disease, Beijing, China

**Keywords:** hyperthyroidism, hypothyroidism, major adverse cardiovascular events, any fracture, sex difference

## Abstract

Objective: To evaluate whether sex differences in the associations of subclinical hypothyroidism (SH) and subclinical hyperthyroidism (SCH) with the risks of major adverse cardiovascular events (MACE) and fractures.

Methods: The PubMed, EmBase, and Cochrane Library databases were searched for eligible studies from inception until November 2021. The relative risk (RR) ratio with the 95% confidence interval (CI) was used to identify sex differences in the associations of SH and SCH with the risks of MACE and fractures. All analyses were performed using a random-effects model.

Results: Twenty-four cohort studies (in 3,480,682 patients) were selected for meta-analysis. There were no sex differences in the associations of SH and SCH with the risks of atrial fibrillation, all-cause mortality, cardiac death, coronary heart disease, heart failure, MACE, stroke, fracture. Subgroup analyses indicated a greater risk of MACE in men than in women with SH if follow-up was ≥10.0 years (RR ratio 2.44; 95% CI 1.17–5.10; *P* = 0.017). The risk of any fracture was greater in men than in women with SH if follow-up was <10.0 years (RR ratio 1.17; 95% CI 1.03–1.34; *P* = 0.017) and in studies with a high level of adjustment (RR ratio 1.16; 95% CI 1.02–1.32; *P* = 0.022). However, the risk of hip fracture was lower in men than in women with SH on pooling of studies with low adjustment (RR ratio 0.53; 95% CI 0.29–0.97; *P* = 0.039).

Conclusions: There may be sex-related differences in the risks of MACE, any fracture, and hip fracture in patients with SH.

## INTRODUCTION

Subclinical thyroid dysfunction (STD) is common in the adult population and is characterized by the absence of symptoms, an abnormal thyroid-stimulating hormone (TSH) level, and normal free thyroxine and free triiodothyronine levels [1]. Reports of the prevalence of subclinical hypothyroidism (SH) have ranged from 4.0% to 20.0% and those of subclinical hyperthyroidism (SCH) from 0.7% to 9.0% [2]. Given the low rate of progression (<0.5%) of STD to overt disease within 5 years in older individuals [3] and the limited amount of evidence to suggest that early treatment alters the clinical course, there is controversy regarding the best treatment for STD. Investigation of the potential impact of STD on long-term adverse health outcomes would help to guide clinical management of individuals at high risk.

Previous studies have already addressed the long-term adverse health outcomes in patients with STD, including cardiovascular disease, stroke, all-cause mortality, chronic kidney disease, cognitive decline, decreased bone mineral density, and fracture. These studies found that STD was associated with an increased risk of long-term adverse health outcomes [4–9]. Sexual dimorphism exists in both thyroid disorders and in health outcomes. However, the sex-specific associations of STD with long-term adverse health outcomes remain unclear. In view of the small number of reports on sex-related differences in associations of subclinical SH and SCH with chronic kidney disease, cognitive decline, and decreased bone mineral density, we performed this systematic review and meta-analysis to assess differences in the association of STD and major adverse cardiovascular events (MACE) and fractures between men and women.

## METHODS

### Data sources, search strategy, and selection criteria

The protocol for reporting a Meta-Analysis of Observational Studies in Epidemiology was used to perform and report this systematic review and meta-analysis [10]. Cohort studies that investigated the associations of SCH or SH with the risk of MACE and fractures according to sex were eligible for inclusion and no restrictions were placed on publication language or publication status. We systematically searched the PubMed, EmBase, and Cochrane Library databases for eligible studies from inception through to November 2021 using the following medical subject headings or text words as search terms: “hypothyroidism,” “subclinical hypothyroidism,” “hyperthyroidism,” “subclinical hyperthyroidism,” “thyroid diseases,” “thyroid function,” “thyroid status,” “cohort studies,” “prospective studies,” and “follow-up studies”. We also manually searched the reference lists of original articles to identify any additional studies that met the inclusion criteria.

The literature search and study selection were performed by two authors working independently. Any disagreement between the authors was resolved by discussion until consensus was reached. Studies were eligible for inclusion if they met the following criteria: (1) participants were a general population for which outcomes had not been previously investigated; (2) exposure and control, that is, SCH or SH and TSH were within the normal range; (3) outcomes included atrial fibrillation (AF), all-cause mortality, cardiac death, coronary heart disease (CHD), heart failure, MACE, stroke, any fracture (at various sites), hip fracture, non-vertebral fracture (including hip fracture and other fractures at non-vertebral sites), and vertebral fracture; (4) sex-based analysis specific for men and women or data stratified by sex; and (5) a cohort study design.

### Data collection and quality assessment

Two authors independently extracted the following information: name of the study group, year of publication, country, design, sample size, patient age and sex, setting, definitions of SCH and SH, use of thyroid medication, outcomes reported, follow-up duration, and adjustment for confounding factors. The same two authors then used the Newcastle–Ottawa Scale to assess the quality of each study based on the three domains of selection, comparability, and outcome. The total scores in each study ranged from 0 to 9 stars, with a score of ≥7 stars indicating a high-quality study. Inconsistencies in data collection and quality assessment were resolved by a third author after consulting the original article.

### Statistical analysis

The associations of SCH or SH with the risk of AF, all-cause mortality, cardiac death, CHD, heart failure, MACEs, stroke, any fracture, hip fracture, non-vertebral fracture, and vertebral fracture were evaluated to estimate the sex-specific effect with its 95% confidence interval (CI). Pooled relative risks (RRs) and 95% CIs were calculated for men and women using a random-effects model [11, 12]. Heterogeneity across the included studies was assessed using the *I*^2^ value and the Q statistic; significant heterogeneity was defined as an *I*^2^ value >50.0% or a *P*-value <0.10 [13]. Next, an indirect comparison of the pooled RRs were performed for men and women, and the RR ratio with the 95% CI was obtained. Sensitivity analysis was performed to evaluate the robustness of the pooled effect estimate of the sex difference after removing studies that reported data only for male or female subjects. Subgroup analyses were performed to detect sex differences based on duration of follow-up and level of adjustment. Reported outcomes that were adjusted for five or more factors were considered to be highly adjusted. Potential publication bias was assessed using a funnel plot with Egger’s test and Begg’s test [14, 15]. All pooled outcomes were two-sided, and a *P*-value <0.05 was considered statistically significant. The statistical analysis was performed using STATA software (version 10.0; StataCorp, College Station, TX, USA).

### Availability of data and materials

All data generated or analyzed during this study are included in this published article.

## RESULTS

### Literature search

**Figure 1:**
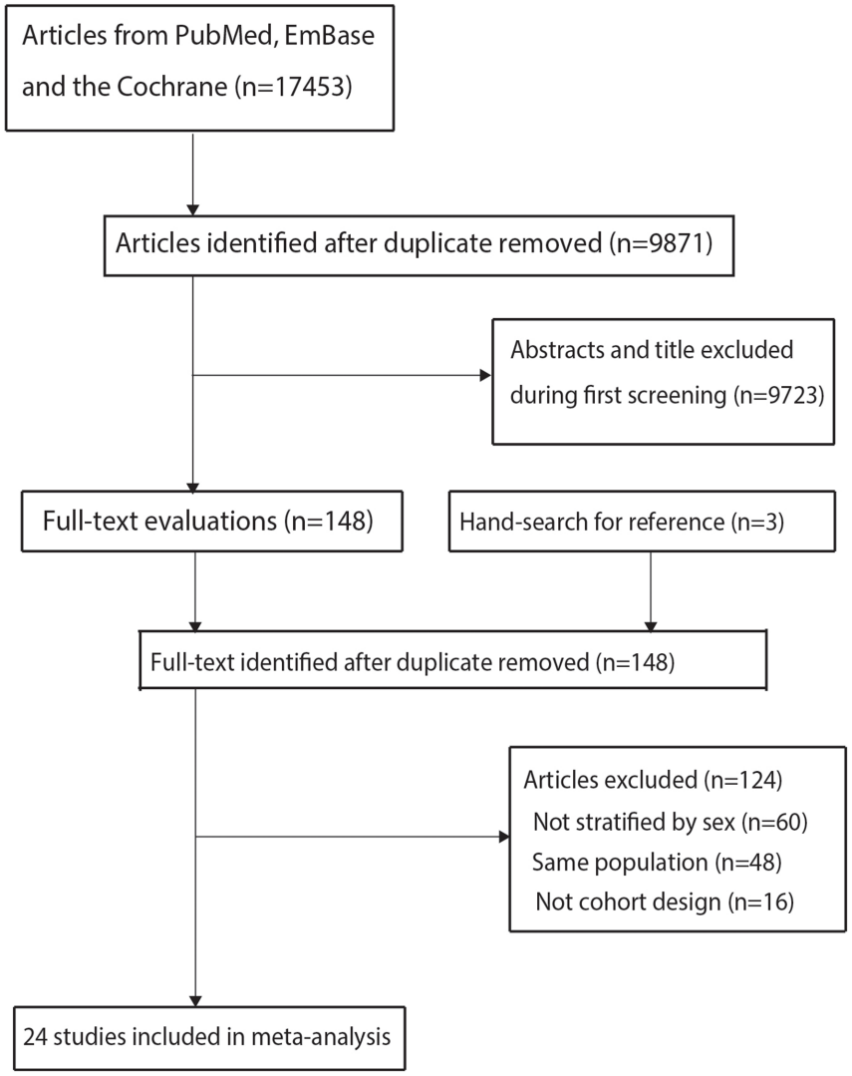
The details of literature search and study selection process.

The initial electronic search produced 17,453 records, 9,871 of which were retained after removing duplicate records. A further 9,723 studies were removed because of irrelevant titles or abstracts. The remaining 148 studies were retrieved for full-text evaluation; 124 of these studies were removed because the subjects were not divided into male and female (*n* = 60), the same population was reported (*n* = 48), or a cohort design was not used (*n* = 16). Review of the reference lists of the relevant studies did not identify any additional eligible studies. Finally, 24 cohort studies [16–39] were selected for meta-analysis (Figure 1).

### Study characteristics

The background characteristics of the study participants are shown in Table 1. The 24 included studies had recruited a total of 3,480,682 individuals. Seventeen studies had a prospective cohort design and the remaining seven studies had a retrospective cohort design. Four studies were performed in Asia, 11 were performed in Europe, and the remaining nine were performed in the US or Australia. The follow-up duration ranged from 1.5 to 28.0 years and each study included 375–1,239,441 participants. The quality of each study is shown in Supplementary Table 1. All of the included studies were of high quality; three had 9 stars, sixteen had 8 stars, and the remaining five had 7 stars.

**Table 1:** The baseline characteristics of included studies.

**Study**	**Country**	**Design**	**Sample size**	**Age (years)**	**Gender**	**Setting**	**Hyperthyroidism**	**Hypothyroidism**	**TM users**	**Reported outcomes**	**Follow-up (years)**	**Adjusted factors**
Rotterdam 2000 [16]	Netherlands	Pro	1,149	>55.0	Females	Population-based	NA	≥4.0	NA	MI	4.6	Age, BMI, cholesterol level, HDL-C, SBP, DBP, and smoking
SOF 2001 [17]	US	Pro	9,704	>65.0	Females	Population-based	<0.50	≥5.5	12.2%	Fracture (hip, vertebral, non- vertebral), cardiac death, all-cause mortality	3.7	TM, previous hyperthyroidism, age, good or excellent self-rated health, current oral estrogen use
Birmingham 2001 [18]	UK	Pro	1,191	>60.0	Males and females	Population-based	<0.50	≥5.0	NA	All-cause mortality, cardiac death	8.2	Age
RERF 2004 [19]	Japan	Pro	2,856	58.5	Males and females	Population-based	NA	≥5.0	NA	MACE, CHD, stroke, all-cause mortality	10.0	Age, smoking
Sheffield 2008 [20]	UK	Pro	375	>50.0	Females	Population-based	<0.45	≥4.5	5.0%	Fracture (hip, non-vertebral)	10.0	Age, smoking, BMI, history of DM, thyroid medication
CHS 2010 [21]	US	Pro	3,576	>65.0	Males and females	Population-based	<0.45	≥4.5	8.3%	Fracture (hip)	12.8	Age, race, self-reported health status, frailty status, smoking, alcohol, height, weight, calcium intake, AOM
OPUS 2010 [22]	Germany, France, England	Pro	1,278	>20.0	Females	Population-based	<0.45	≥4.5	0.0%	Fracture (hip, non-vertebral)	6.0	Thyroid and thyroid-altering medication, AOM
PROSPER 2012 [23]	Netherlands, Scotland, Ireland	Pro	5,316	>70.0	Males and females	Population-based	<0.45	NA	2.5%	Heart failure	3.2	Age, sex, education, history of CVD, DM, BMI, smoking, SBP, LDL-C, creatinine, and β-blocker and antiarrhythmic use
DNPR 2012 [24]	Denmark	Retro	586,460	>18.0	Males and females	Population-based	<0.45	≥4.5	NA	Atrial fibrillation, all-cause mortality, MACE, MI, heart failure, stroke	5.5	Sex, age, calendar year, Charlson comorbidity index, and socioeconomic status
MrOS-US 2013 [25]	US	Pro	1,588	>65.0	Males	Population-based	<0.55	≥4.78	7.6%	Fracture (hip, any, vertebral, non-vertebral), cardiac death, all-cause mortality	11.1	Age, clinic site, race, BMI, PA score, alcohol, smoking, corticosteroid use, and TM
HUNT 2013 [26]	Norway	Pro	25,205	>40.0	Males and females	Population-based	TSH <0.50	>3.5	4.7%	Fracture (hip, any, non-vertebral), all-cause mortality, cardiac death, MI, heart failure	12.5	Age, BMI, and smoking
WHI-OS 2013 [27]	US	Pro	93,676	>50.0	Females	Population-based	NA	>4.68	NA	MI, stroke	>5.0	Age, ethnicity, gravidity, smoking, hormone therapy, and alcohol
OPENTHYRO 2014 [28]	Denmark	Retro	231,355	>18.0	Males and females	Population-based	<0.30	>4.0	NA	Fracture (hip, any)	7.5	Age, sex, Charlson index, prednisolone in last year, AOM, DM, dementia, CHF, malignancy, liver disease, rheumatic diseases, pulmonary disease, major osteoporotic fracture, and year
Ansung cohort study 2014 [29]	Korea	Pro	2,968	>40.0	Males and females	Population-based	NA	>4.0	NA	MACE	10.0	Reynolds risk score
General Hospital Vienna 2015 [30]	Austria	Retro	80,490	>18.0	Males and females	Population-based	NA	≥4.5	NA	All-cause mortality	4.1	Age
LDO 2015 [31]	US	Retro	8,840	>18.0	Males and females	Population-based	NA	>5.0	NA	All-cause mortality	1.5	Entry quarter, age, sex, race/ethnicity, cause of end-stage renal disease, vascular access, dialysis vintage, BMI, DM, CHF, cerebrovascular disease, MI, other cardiac disease, hypertension, and PAD
USRT 2017 [32]	US	Pro	75,056	>20.0	Females	Population-based	<0.40	>4.0	NA	Cardiac death, stroke	28.0	Baseline year and age, race/ethnicity, BMI, family history of breast cancer, lifestyle and reproductive factors
HIMS 2018 [33]	Australia	Pro	4,248	>70.0	Males	Population-based	<0.40	>4.0	NA	Fracture (hip), all-cause mortality	3.5	Age, smoking, BMI, waist-hip ratio, alcohol, PA, hypertension, dyslipidaemia, DM, CVD, cancer, frailty, creatinine and vitamin D
US veterans 2018 [34]	US	Pro	227,422	71.0	Males and females	Population-based	<0.50	>5.0	6.0%	All-cause mortality	5.0	Age, sex, race, ethnicity, DM, CHF, CVD, hypertension, hyperlipidemia, and Charlson comorbidity index
Taiwan NHI 2018 [35]	China	Retro	4,540	>20.0	Males and females	Population-based	NA	≥4.5	NA	All-cause mortality, stroke	3.0	Sex, age, DM, hyperlipidemia, hypertension, CVD, CHF, stroke, PAD, chronic obstructive pulmonary disease, asthma, and cancer
THIN 2019 [36]	UK	Retro	863,072	>18.0	Males and females	Population-based	<0.40	>4.0	81.7%	Fracture (any), CHD, heart failure, stroke, atrial fibrillation, all-cause mortality	6.0	Age, sex, BMI, smoking, fifth of townsend deprivation, prescription for lipid lowering drug, DM, hypertension, and prescription for levothyroxine
NHANES 2020 [37]	US	Pro	9,020	>20.0	Males and females	Population-based	NA	>5.6	NA	All-cause mortality	7.3	Age, race/ethnicity, education status, smoking, cancer history, and estimated glomerular filtration rate
Korean NHI 2020 [38]	Korea	Retro	1,239,441	>20.0	Males and females	Population-based	<0.45	NA	NA	MI, stroke	10.0	Hypertension, fasting glucose, smoking, cholesterol, and obesity
MrOS-Sweden 2021 [39]	Sweden	Pro	1,856	>69.0	Males	Population-based	<0.45	NA	1.7%	Fracture (any, vertebral)	8.9	Age, MrOS site, levothyroxine treatment, BMI, appendicular lean mass, grip strength, walking speed, smoking, and total hip sBMD

Abbreviations: AOM: antiosteoporosis medication; BMD: bone mineral density; BMI: body mass index; CHF: congestive heart failure; CVD: cardiovascular disease; DBP: diastolic blood pressure; DM: diabetes mellitus; HDL-C: high-density lipoprotein cholesterol; LDL-C: low-density lipoprotein cholesterol; PA: physical activity; PAD: peripheral arterial disease; Pro: prospective; Retro: retrospective; SBP: systolic blood pressure; TM: thyroid medication.

### Atrial fibrillation

**Figure 2:**
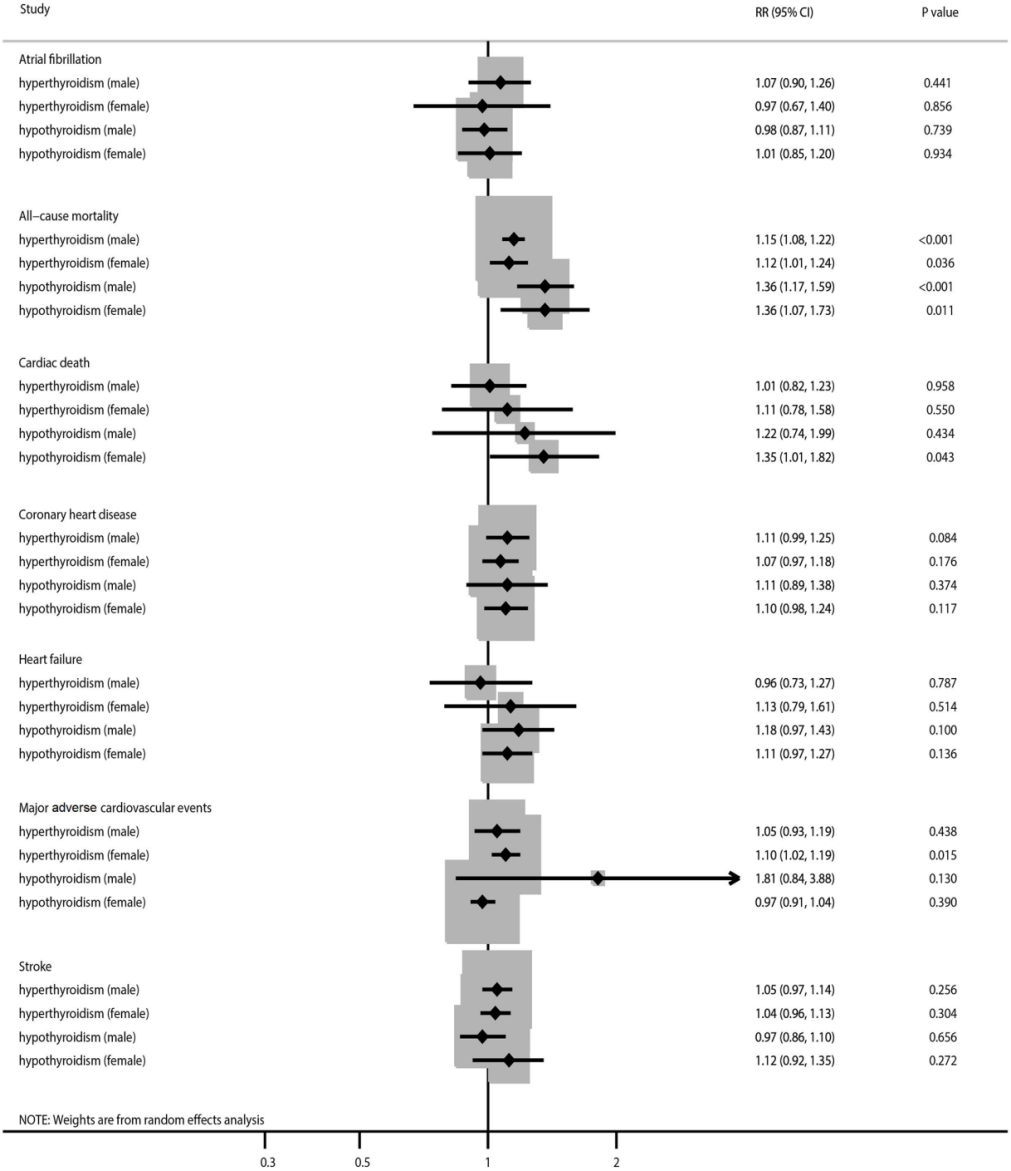
The summary results stratified by gender in the association of subclinical thyroid dysfunction with the risk of atrial fibrillation, all-cause mortality, cardiac death, coronary heart disease, heart failure, major adverse cardiovascular events, and stroke.

SCH and SH were not associated with an increased risk of AF irrespective of sex (Figure 2, Supplementary Figures 1 and 2). Moreover, we noted significant heterogeneity in the association of SCH or SH with the risk of AF in women. There were no significant sex-related differences in the association of SCH (RR ratio 1.10; 95% CI 0.74–1.66; *P* = 0.636) or SH (RR ratio 0.97; 95% CI 0.79–1.20; *P* = 0.780) with the risk of AF (Table 2). These findings persisted after removing indirect comparison studies (Table 3). The results of subgroup analyses were consistent with those of the overall analysis, and any sex-related differences in the association of SCH or SH with the risk of AF were not significant (Table 4). No significant publication bias with regard to the association of SCH or SH with the risk of AF was observed (Supplementary Figures 3 and 4).

**Table 2:** Sex difference in long term health outcomes for patients with subclinical thyroid dysfunction.

**Outcomes**	**Subclinical thyroid dysfunction**	**Males**	**Females**	**RR ratio**	***P* value**
Atrial fibrillation	Hyperthyroidism	1.07 (0.90–1.26)	0.97 (0.66–1.40)	1.10 (0.74–1.66)	0.636
	Hypothyroidism	0.98 (0.87–1.11)	1.01 (0.85–1.20)	0.97 (0.79–1.20)	0.780
All-cause mortality	Hyperthyroidism	1.15 (1.08–1.22)	1.12 (1.01–1.24)	1.03 (0.91–1.16)	0.664
	Hypothyroidism	1.36 (1.17–1.59)	1.36 (1.07–1.73)	1.00 (0.75–1.33)	1.000
Cardiac death	Hyperthyroidism	1.01 (0.82–1.23)	1.11 (0.78–1.58)	0.91 (0.61–1.37)	0.649
	Hypothyroidism	1.22 (0.74–1.99)	1.35 (1.01–1.82)	0.90 (0.51–1.61)	0.730
Coronary heart disease	Hyperthyroidism	1.11 (0.99–1.25)	1.07 (0.97–1.18)	1.04 (0.89–1.21)	0.637
	Hypothyroidism	1.11 (0.89–1.38)	1.10 (0.98–1.24)	1.01 (0.79–1.29)	0.943
Heart failure	Hyperthyroidism	0.96 (0.73–1.27)	1.13 (0.79–1.61)	0.85 (0.54–1.33)	0.479
	Hypothyroidism	1.18 (0.97–1.43)	1.11 (0.97–1.27)	1.06 (0.84–1.35)	0.612
Major advance cardiovascular events	Hyperthyroidism	1.05 (0.93–1.19)	1.10 (1.02–1.19)	0.96 (0.83–1.10)	0.531
	Hypothyroidism	1.81 (0.84–3.88)	0.97 (0.91–1.04)	1.87 (0.87–4.02)	0.111
Stroke	Hyperthyroidism	1.05 (0.97–1.14)	1.04 (0.96–1.13)	1.01 (0.90–1.13)	0.870
	Hypothyroidism	0.97 (0.86–1.10)	1.12 (0.92–1.35)	0.87 (0.69–1.09)	0.216
Any fracture	Hyperthyroidism	1.17 (1.00–1.36)	1.02 (0.93–1.12)	1.15 (0.96–1.37)	0.134
	Hypothyroidism	1.10 (0.96–1.26)	0.99 (0.94–1.05)	1.11 (0.96–1.29)	0.160
Hip fracture	Hyperthyroidism	1.47 (1.04–2.07)	1.38 (1.01–1.89)	1.07 (0.67–1.70)	0.790
	Hypothyroidism	0.97 (0.67–1.41)	0.97 (0.89–1.06)	1.00 (0.68–1.47)	1.000
Non-vertebral fracture	Hyperthyroidism	1.18 (0.45–3.11)	1.24 (0.87–1.77)	0.95 (0.34–2.66)	0.925
	Hypothyroidism	0.96 (0.63–1.46)	1.31 (0.79–2.16)	0.73 (0.38–1.41)	0.353
Vertebral fracture	Hyperthyroidism	3.07 (1.62–5.79)	3.43 (1.52–7.71)	0.90 (0.32–2.51)	0.833

**Table 3:** Sensitivity analysis for direct comparisons of sex difference in long term health outcomes for patients with subclinical thyroid dysfunction.

**Outcomes**	**Subclinical thyroid dysfunction**	**Males**	**Females**	**RR ratio**	***P* value**
Atrial fibrillation	Hyperthyroidism	1.07 (0.90–1.26)	0.97 (0.66–1.40)	1.03 (0.84–1.25)	0.797
	Hypothyroidism	0.98 (0.87–1.11)	1.01 (0.85–1.20)	0.99 (0.85–1.14)	0.848
All-cause mortality	Hyperthyroidism	1.15 (1.09–1.23)	1.12 (1.01–1.25)	1.01 (0.93–1.09)	0.876
	Hypothyroidism	1.39 (1.18–1.64)	1.43 (1.09–1.86)	0.97 (0.90–1.04)	0.344
Cardiac death	Hyperthyroidism	1.00 (0.80–1.20)	0.90 (0.70–1.10)	1.11 (0.82–1.51)	0.496
	Hypothyroidism	1.06 (0.65–1.72)	1.76 (1.21–2.56)	0.60 (0.33–1.11)	0.106
Coronary heart disease	Hyperthyroidism	1.11 (0.99–1.25)	1.07 (0.97–1.18)	1.01 (0.86–1.19)	0.878
	Hypothyroidism	1.11 (0.89–1.38)	1.06 (0.98–1.16)	0.99 (0.86–1.16)	0.945
Heart failure	Hyperthyroidism	0.96 (0.73–1.27)	1.06 (0.71–1.57)	0.96 (0.74–1.24)	0.751
	Hypothyroidism	1.18 (0.97–1.43)	1.11 (0.97–1.27)	1.08 (0.94–1.24)	0.268
Major advance cardiovascular events	Hyperthyroidism	1.05 (0.93–1.19)	1.10 (1.02–1.19)	0.95 (0.83–1.10)	0.531
	Hypothyroidism	1.81 (0.84–3.88)	0.97 (0.91–1.04)	1.59 (0.81–3.12)	0.181
Stroke	Hyperthyroidism	1.05 (0.97–1.14)	1.04 (0.95–1.14)	0.97 (0.88–1.08)	0.620
	Hypothyroidism	0.97 (0.86–1.10)	1.10 (0.87–1.40)	1.02 (0.87–1.18)	0.841
Any fracture	Hyperthyroidism	1.10 (0.94–1.30)	1.02 (0.93–1.12)	1.07 (0.90–1.28)	0.423
	Hypothyroidism	1.10 (0.94–1.29)	0.99 (0.94–1.05)	1.10 (0.92–1.33)	0.302
Hip fracture	Hyperthyroidism	1.34 (0.86–2.09)	1.15 (0.98–1.34)	1.13 (0.81–1.58)	0.473
	Hypothyroidism	0.88 (0.56–1.37)	0.97 (0.88–1.07)	0.87 (0.55–1.39)	0.565
Non-vertebral fracture	Hyperthyroidism	0.47 (0.07–3.41)	1.08 (0.75–1.56)	0.44 (0.06–3.14)	0.410
	Hypothyroidism	0.89 (0.40–1.98)	1.11 (0.90–1.37)	0.80 (0.35–1.83)	0.601

**Table 4:** Subgroup analyses for investigated outcomes.

**Outcomes**	**Factors**	**Subgroups**	**Males**	**Females**	**RR ratio**	***P* value**
Atrial fibrillation (hyperthyroidism)	Follow-up (years)	≥10.0	−	−	−	−
		<10.0	1.07 (0.90–1.26)	0.97 (0.66–1.40)	1.10 (0.73–1.67)	0.641
	Adjusted level	High	0.95 (0.77–1.18)	0.81 (0.71–0.92)	1.17 (0.91–1.51)	0.211
		Low	1.20 (0.99–1.45)	1.35 (1.20–1.51)	0.89 (0.71–1.11)	0.300
Atrial fibrillation (hypothyroidism)	Follow-up (years)	≥10.0	−	−	−	−
		<10.0	0.98 (0.87–1.11)	1.01 (0.85–1.20)	0.97 (0.79–1.20)	0.780
	Adjusted level	High	1.01 (0.86–1.18)	1.09 (0.99–1.21)	0.93 (0.77–1.12)	0.425
		Low	0.94 (0.77–1.14)	0.86 (0.77–0.97)	1.09 (0.87–1.37)	0.444
All-cause mortality (hyperthyroidism)	Follow-up (years)	≥10.0	0.78 (0.41–1.48)	−	−	−
		<10.0	1.15 (1.09–1.23)	1.12 (1.01–1.24)	1.03 (0.91–1.16)	0.663
	Adjusted level	High	1.12 (1.00–1.25)	1.14 (1.02–1.28)	0.98 (0.84–1.15)	0.827
		Low	1.17 (1.06–1.29)	1.06 (0.79–1.42)	1.10 (0.81–1.50)	0.531
All-cause mortality (hypothyroidism)	Follow-up (years)	≥10.0	1.14 (0.83–1.58)	1.10 (0.69–1.76)	1.04 (0.59–1.83)	0.902
		<10.0	1.34 (1.15–1.57)	1.45 (1.11–1.89)	0.92 (0.68–1.26)	0.616
	Adjusted level	High	1.31 (1.12–1.53)	1.49 (1.16–1.91)	0.88 (0.66–1.18)	0.391
		Low	1.44 (0.75–2.74)	1.25 (0.71–2.21)	1.15 (0.49–2.73)	0.747
Cardiac death (hyperthyroidism)	Follow-up (years)	≥10.0	1.12 (0.45–2.78)	1.43 (0.62–2.31)	0.78 (0.25–2.41)	0.670
		<10.0	1.00 (0.82–1.22)	0.90 (0.72–1.12)	1.11 (0.83–1.50)	0.487
	Adjusted level	High	1.12 (0.45–2.78)	0.97 (0.73–1.28)	1.15 (0.45–2.99)	0.767
		Low	1.00 (0.82–1.22)	1.38 (0.55–3.43)	0.72 (0.28–1.85)	0.500
Cardiac death (hypothyroidism)	Follow-up (years)	≥10.0	1.22 (0.74–1.99)	1.40 (0.98–2.01)	0.87 (0.47–1.61)	0.659
		<10.0	−	0.92 (0.25–3.36)	−	−
	Adjusted level	High	1.73 (0.49–6.09)	1.21 (1.03–1.41)	1.43 (0.40–5.09)	0.581
		Low	1.06 (0.65–1.72)	1.76 (1.21–2.56)	0.60 (0.33–1.11)	0.106
Coronary heart disease (hyperthyroidism)	Follow-up (years)	≥10.0	1.33 (0.84–2.09)	1.20 (1.02–1.42)	1.11 (0.68–1.80)	0.678
		<10.0	1.05 (0.90–1.23)	1.02 (0.91–1.14)	1.03 (0.85–1.25)	0.768
	Adjusted level	High	1.09 (0.96–1.24)	1.10 (0.96–1.27)	0.99 (0.82–1.20)	0.925
		Low	1.32 (0.79–2.18)	0.99 (0.84–1.17)	1.33 (0.78–2.27)	0.291
Coronary heart disease (hypothyroidism)	Follow-up (years)	≥10.0	1.75 (0.46–6.58)	1.17 (0.85–1.62)	1.50 (0.38–5.88)	0.564
		<10.0	1.07 (0.87–1.31)	1.10 (0.95–1.26)	0.97 (0.76–1.25)	0.827
	Adjusted level	High	1.05 (0.74–1.49)	1.11 (0.90–1.37)	0.95 (0.63–1.42)	0.790
		Low	1.23 (0.81–1.89)	1.13 (0.99–1.28)	1.09 (0.70–1.69)	0.707
Heart failure (hyperthyroidism)	Follow-up (years)	≥10.0	−	1.65 (0.89–3.07)	−	−
		<10.0	0.96 (0.73–1.27)	1.06 (0.71–1.57)	0.91 (0.56–1.47)	0.688
	Adjusted level	High	0.88 (0.57–1.34)	0.99 (0.61–1.60)	0.89 (0.47–1.69)	0.720
		Low	1.10 (0.93–1.30)	1.25 (1.13–1.39)	0.88 (0.72–1.07)	0.203
Heart failure (hypothyroidism)	Follow-up (years)	≥10.0	0.62 (0.26–1.47)	1.15 (0.86–1.54)	0.54 (0.22–1.34)	0.185
		<10.0	1.21 (1.01–1.45)	1.11 (0.94–1.31)	1.09 (0.85–1.39)	0.491
	Adjusted level	High	1.30 (0.96–1.75)	1.20 (0.96–1.49)	1.08 (0.75–1.57)	0.673
		Low	0.98 (0.61–1.57)	1.01 (0.91–1.11)	0.97 (0.60–1.57)	0.903
Major advance cardiovascular events (hyperthyroidism)	Follow-up (years)	≥10.0	−	−	−	−
		<10.0	1.05 (0.93–1.19)	1.10 (1.02–1.19)	0.95 (0.83–1.10)	0.531
	Adjusted level	High	−	−	−	−
		Low	1.05 (0.93–1.19)	1.10 (1.02–1.19)	0.95 (0.83–1.10)	0.531
Major advance cardiovascular events (hypothyroidism)	Follow-up (years)	≥10.0	2.54 (1.42–4.52)	1.04 (0.66–1.64)	**2.44 (1.17–5.10)**	**0.017**
		<10.0	1.03 (0.91–1.17)	0.97 (0.91–1.04)	1.06 (0.92–1.22)	0.408
	Adjusted level	High	−	−	−	−
		Low	1.81 (0.84–3.88)	0.97 (0.91–1.04)	1.87 (0.87–4.02)	0.111
Stroke (hyperthyroidism)	Follow-up (years)	≥10.0	1.05 (0.95–1.16)	1.13 (1.04–1.22)	0.93 (0.82–1.06)	0.260
		<10.0	1.05 (0.91–1.22)	1.00 (0.90–1.11)	1.05 (0.88–1.26)	0.596
	Adjusted level	High	1.07 (0.97–1.17)	1.04 (0.93–1.16)	1.03 (0.89–1.19)	0.700
		Low	0.98 (0.80–1.20)	1.03 (0.92–1.15)	0.95 (0.75–1.20)	0.673
Stroke (hypothyroidism)	Follow-up (years)	≥10.0	0.60 (0.11–3.17)	1.28 (0.93–1.75)	0.47 (0.08–2.59)	0.385
		<10.0	0.97 (0.86–1.10)	1.09 (0.88–1.35)	0.89 (0.70–1.14)	0.354
	Adjusted level	High	0.99 (0.83–1.17)	1.17 (0.89–1.55)	0.85 (0.61–1.17)	0.316
		Low	0.95 (0.79–1.15)	0.93 (0.84–1.03)	1.02 (0.83–1.26)	0.845
Any fracture (hyperthyroidism)	Follow-up (years)	≥10.0	1.16 (0.62–2.18)	1.14 (0.86–1.51)	1.02 (0.51–2.03)	0.961
		<10.0	1.17 (0.98–1.41)	1.00 (0.91–1.11)	1.17 (0.95–1.44)	0.138
	Adjusted level	High	1.18 (1.00–1.40)	1.00 (0.91–1.11)	1.18 (0.97–1.43)	0.097
		Low	0.84 (0.37–1.90)	1.14 (0.86–1.51)	0.74 (0.31–1.75)	0.489
Any fracture (hypothyroidism)	Follow-up (years)	≥10.0	0.85 (0.61–1.17)	1.10 (0.93–1.30)	0.77 (0.54–1.11)	0.168
		<10.0	1.15 (1.02–1.30)	0.98 (0.93–1.03)	**1.17 (1.03–1.34)**	**0.017**
	Adjusted level	High	1.14 (1.01–1.28)	0.98 (0.93–1.03)	**1.16 (1.02–1.32)**	**0.022**
		Low	0.72 (0.46–1.13)	1.10 (0.93–1.30)	0.65 (0.41–1.06)	0.083
Hip fracture (hyperthyroidism)	Follow-up (years)	≥10.0	1.98 (0.87–4.50)	1.59 (0.79–3.21)	1.25 (0.42–3.67)	0.691
		<10.0	1.30 (0.96–1.76)	1.41 (0.85–2.32)	0.92 (0.51–1.66)	0.786
	Adjusted level	High	1.64 (1.06–2.53)	1.70 (0.97–2.97)	0.96 (0.47–1.96)	0.921
		Low	0.99 (0.40–2.44)	1.28 (0.86–1.90)	0.77 (0.29–2.08)	0.610
Hip fracture (hypothyroidism)	Follow-up (years)	≥10.0	1.18 (0.53–2.65)	1.11 (0.89–1.37)	1.06 (0.46–2.45)	0.886
		<10.0	0.79 (0.57–1.10)	0.94 (0.85–1.04)	0.84 (0.60–1.19)	0.322
	Adjusted level	High	1.06 (0.68–1.65)	0.94 (0.85–1.03)	1.13 (0.72–1.77)	0.604
		Low	0.64 (0.37–1.10)	1.20 (0.94–1.53)	**0.53 (0.29–0.97)**	**0.039**
Non-vertebral fracture (hyperthyroidism)	Follow-up (years)	≥10.0	1.18 (0.45–3.11)	1.14 (0.80–1.61)	1.04 (0.37–2.89)	0.948
		<10.0	−	1.42 (0.71–2.83)	−	−
	Adjusted level	High	1.54 (0.63–3.76)	2.05 (1.17–3.61)	0.75 (0.26–2.16)	0.595
		Low	0.47 (0.07–3.28)	0.99 (0.74–1.34)	0.47 (0.07–3.32)	0.453
Non-vertebral fracture (hypothyroidism)	Follow-up (years)	≥10.0	0.96 (0.63–1.46)	1.38 (0.78–2.45)	0.70 (0.34–1.41)	0.316
		<10.0	−	0.38 (0.02–6.69)	−	−
	Adjusted level	High	0.99 (0.60–1.62)	2.05 (1.04–4.05)	0.48 (0.21–1.12)	0.090
		Low	0.89 (0.40–1.98)	1.10 (0.90–1.36)	0.81 (0.35–1.85)	0.615
Vertebral fracture (hyperthyroidism)	Follow-up (years)	≥10.0	1.29 (0.18–9.32)	−	−	−
		<10.0	3.39 (1.73–6.64)	3.43 (1.52–7.71)	0.99 (0.34–2.84)	0.983
	Adjusted level	High	3.07 (1.62–5.79)	3.43 (1.52–7.71)	0.90 (0.32–2.51)	0.833
		Low	−	−	−	−

### All-cause mortality

SCH and SH were associated with an increased risk of all-cause mortality in both men and women (Figure 2, Supplementary Figures 5 and 6). There was significant heterogeneity for all-cause mortality related to SCH in women and all-cause mortality related to SH in both sexes. No sex differences were observed in the association of SCH (RR ratio 1.03; 95% CI 0.91–1.16; *P* = 0.664) or SH (RR ratio 1.00; 95% CI 0.75–1.33; *P* = 1.000) with the risk of all-cause mortality (Table 2). The pooled conclusions for these associations were robust (Table 3). The results of the subgroup analyses were consistent with those of the overall analysis (Table 4). There was no significant publication bias with regard to studies of the association of SCH or SH with the risk of all-cause mortality (Supplementary Figures 7 and 8).

### Cardiac death

An association was found between SH and an increased risk of cardiac death in women (Figure 2, Supplementary Figures 9 and 10). Moreover, there was significant heterogeneity in terms of cardiac death related to SCH. There were no significant sex-related differences between in the association of SCH (RR ratio 0.91; 95% CI 0.61–1.37; *P* = 0.649) or SH (RR ratio 0.90; 95% CI 0.51–1.61; *P* = 0.730) with the risk of cardiac death (Table 2). Sensitivity analysis indicated that the pooled conclusion for these associations was stable (Table 3). The results of the subgroup analyses were consistent with those of the overall analysis in all subgroups (Table 4). No significant publication bias was detected with regard to studies of the association of SCH or SH with the risk of cardiac death (Supplementary Figures 11 and 12).

### Coronary heart disease

There was no significant association of SCH or SH with the risk of CHD, irrespective of sex (Figure 2, Supplementary Figures 13 and 14). We noted significant heterogeneity in the association of SH with the risk of CHD. No sex differences were observed in the association of SCH (RR ratio 1.04; 95% CI 0.89–1.21; *P* = 0.637) or SH (RR ratio 1.01; 95% CI 0.79–1.29; *P* = 0.943) with the risk of CHD (Table 2). The results of the sensitivity and subgroup analyses were consistent with those of the overall analysis, with no sex differences detected (Tables 3 and 4). However, there was potential publication bias in the association of SH with the risk of CHD (*P*-value for Egger’s test, 0.041; *P*-value for Begg’s test, 0.244; Supplementary Figures 15 and 16).

### Heart failure

We did not find a significant association of SCH or SH with the risk of heart failure regardless of sex (Figure 2, Supplementary Figures 17 and 18). There was potentially significant heterogeneity for heart failure related to SCH or SH in both sexes. There were no significant sex-related differences in the association of SCH (RR ratio 0.85; 95% CI 0.54–1.33; *P* = 0.479) or SH (RR ratio 1.06; 95% CI 0.84–1.35; *P* = 0.612) with the risk of heart failure (Table 2). The results of the sensitivity and subgroup analyses were consistent with those of the overall analysis (Tables 3 and 4). There was no significant publication bias in terms of studies of the association of SCH or SH with the risk of heart failure (Supplementary Figures 19 and 20).

### Major adverse cardiovascular events

SCH was associated with an increased risk of MACE in women and there was potentially significant heterogeneity for MACE related to SH in men (Figure 2, Supplementary Figures 21 and 22). There were no significant sex-related differences in the association of SCH (RR ratio 0.96; 95% CI 0.83–1.10; *P* = 0.531) or SH (RR ratio 1.87; 95% CI 0.87–4.02; *P* = 0.111) with the risk of MACE (Table 2). Sensitivity analysis indicated that the pooled conclusion regarding a sex-related difference was robust after removing the results of indirect comparisons (Table 3). Subgroup analysis found that men with SH had a higher risk of MACE when the follow-up duration was ≥10.0 years (RR ratio 2.44; 95% CI 1.17–5.10; *P* = 0.017; Table 4). No significant publication bias was detected with regard to the association of SH with the risk of MACE (Supplementary Figure 23).

### Stroke

There was no significant association of SCH or SH with the risk of stroke regardless of sex (Figure 2, Supplementary Figures 24 and 25). No sex differences were observed in the association of SCH (RR ratio 1.01; 95% CI 0.90–1.13; *P* = 0.870) or SH (RR ratio 0.87; 95% CI 0.69–1.09; *P* = 0.216) with the risk of stroke (Table 2). The results of the sensitivity and subgroup analyses were consistent with those of the overall analysis (Tables 3 and 4). There was no significant publication bias in terms of the association of SCH or SH with the risk of stroke (Supplementary Figures 26 and 27).

### Any fracture

**Figure 3:**
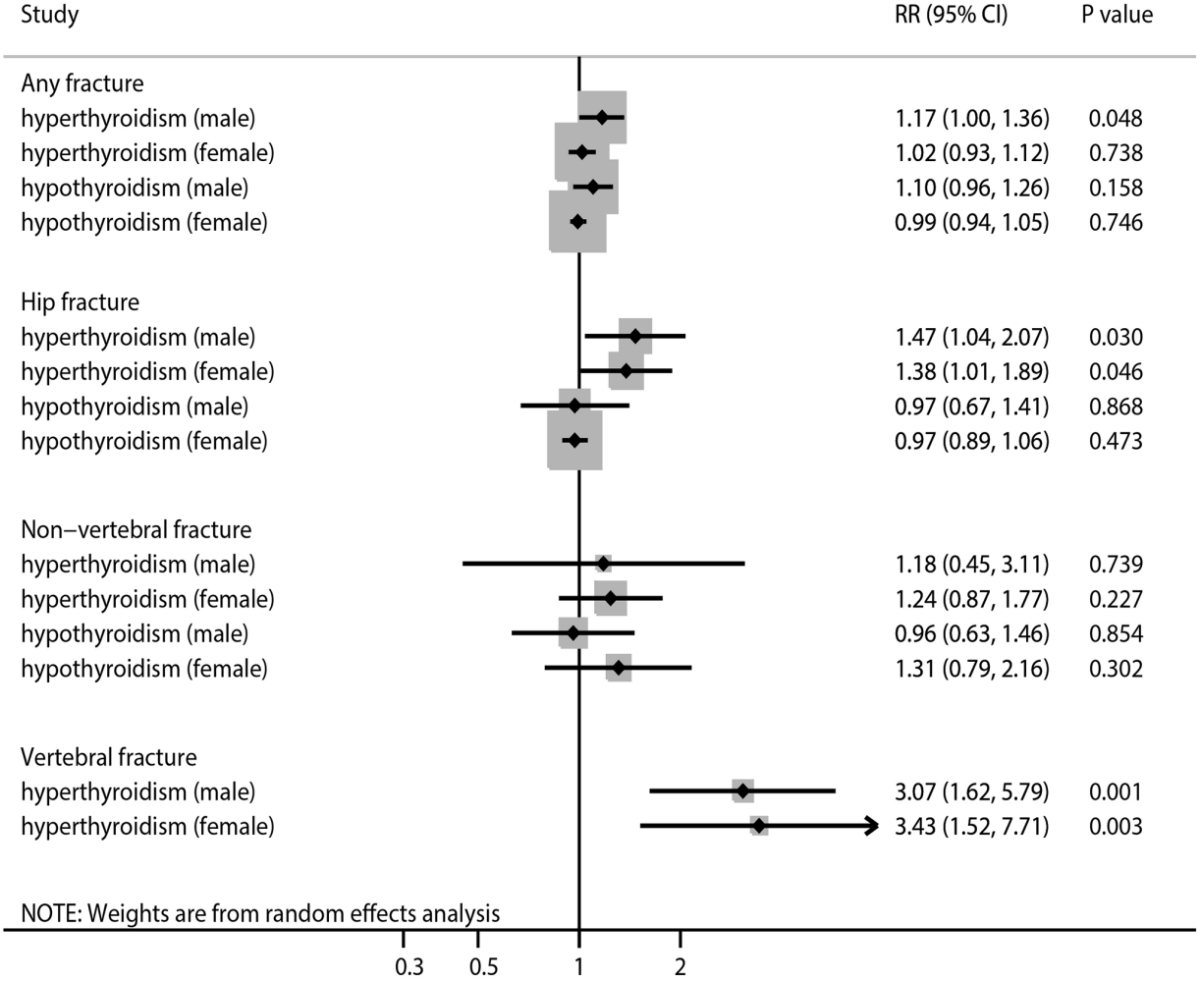
The summary results stratified by gender in the association of subclinical thyroid dysfunction with the risk of any fracture, hip fracture, non-vertebral fracture, and vertebral fracture.

We found an association of SCH with an increased risk of any fracture in men, with no significant heterogeneity observed (Figure 3, Supplementary Figures 28 and 29). Although the risk of any fracture with SCH (RR ratio 1.15; 95% CI 0.96–1.37; *P* = 0.134) or SH (RR ratio 1.11; 95% CI 0.96–1.29; *P* = 0.160) was higher in men, the sex differences were not statistically significant (Table 2). The results of the sensitivity analysis were consistent with those of the overall analysis (Table 3). Subgroup analysis indicated that the risk of any fracture was higher in men with SH than in women with SH when the follow-up duration was <10.0 years (RR ratio 1.17; 95% CI 1.03–1.34; *P* = 0.017) and in studies with a high level of adjustment (RR ratio 1.16; 95% CI 1.02–1.32; *P* = 0.022; Table 4). There was no significant publication bias with regard to the association of SCH or SH with the risk of any fracture (Supplementary Figures 30 and 31).

### Hip fracture

The results indicate that SCH was associated with an increased risk of hip fracture in both men and women, and there was potentially significant heterogeneity for hip fracture related to SH in men (Figure 3, Supplementary Figures 32 and 33). There were no significant differences in the association of SCH (RR ratio 1.07; 95% CI 0.67–1.70; *P* = 0.790) or SH (RR ratio 1.00; 95% CI 0.68–1.47; *P* = 1.000) with the risk of hip fracture between men and women (Table 2). The results of the sensitivity analysis were consistent with those of the overall analysis (Table 3). Subgroup analysis found that the risk of hip fracture was lower in men with SH than in women with SH on pooling of studies with a low level of adjustment (Table 4). There was significant publication bias in terms of the association of SCH with the risk of hip fracture (Supplementary Figures 34 and 35).

### Non-vertebral fracture

There was no significant association of SCH or SH with the risk of non-vertebral fracture in men or women (Figure 3, Supplementary Figures 36 and 37). No sex differences were observed in the association of SCH (RR ratio 0.95; 95% CI 0.34–2.66; *P* = 0.925) or SH (RR ratio 0.73; 95% CI 0.38–1.41; *P* = 0.353) with the risk of non-vertebral fracture (Table 2). The results of sensitivity and subgroup analyses were consistent with those of the overall analysis (Tables 3 and 4). No significant publication bias was detected with regard to the association of SCH or SH with the risk of non-vertebral fracture (Supplementary Figures 38 and 39).

### Vertebral fracture

The results indicate that SCH was associated with an increased risk of vertebral fracture in both men and women, with no evidence of heterogeneity across the included studies (Figure 3, Supplementary Figure 40). There was no significant difference in the association of SCH with the risk of vertebral fracture between men and women (RR ratio 0.90; 95% CI 0.32–2.51; *P* = 0.833; Table 2). The results of the sensitivity and subgroup analyses were consistent with those of the overall analysis (Tables 3 and 4). There was no significant publication bias in terms of the association between SCH and vertebral fracture risk (Supplementary Figure 41).

## DISCUSSION

This meta-analysis of 24 cohort studies reporting data on 3,480,682 individuals found no sex-related differences in the association of SCH or SH with the risk of AF, all-cause mortality, cardiac death, CHD, heart failure, MACE, stroke, any fracture, hip fracture, non-vertebral fracture, or vertebral fracture. Our conclusions were stable in sensitivity analyses. Subgroup analyses found that men with SH had an excess risk of MACE when compared with women if the duration of follow-up was ≥10.0 years. Moreover, the risk of any fracture was greater in men with SH than in women with SH when the follow-up duration was <10.0 years and in studies with a high level of adjustment. In studies with a low level of adjustment, the risk of hip fracture was greater in women with SH than in men with SH.

Although no systematic review and meta-analysis has investigated sex differences in the associations of STD with long-term health outcomes, several studies have assessed these associations with stratification according to sex. A meta-analysis by Collet et al. identified six such studies and found that the risk of CHD was higher in women with SH than in men with SH [40]. Baumgartner et al. analyzed data from 11 cohorts and found that women with SH had an excess risk of AF when compared with their male counterparts [41]. Blum et al. performed a meta-analysis of 13 studies and found that the risks of hip fracture, any fracture, non-vertebral fracture, and vertebral fracture were greater in men with SCH than in women with SCH [42]. However, the above studies did not address the heterogeneity across included studies and did not compare the effect estimates between men and women. Therefore, we performed this systematic review and meta-analysis to determine if there are sex differences in the association of STD with MACE and fractures.

There were no significant differences in the association of STD with the risk of AF, all-cause mortality, cardiac death, CHD, heart failure, MACE, or stroke. However, subgroup analysis found that the risk of MACE was greater in men with SH than in women with SH if the duration of follow-up was ≥10.0 years. A potential reason for this finding could be the high prevalence of metabolically unhealthy obese phenotypes in men with SH, regardless of age. However, metabolically unhealthy non-obese and obese phenotypes were found not to be associated with the risk of SH in women [43]. Moreover, the longer follow-up duration allowed us to obtain more cases of MACE, and the power was sufficient to detect a potential sex difference in the associations of SH with the risk of MACE.

Our results indicate no significant sex-related differences in the association of STD with the risk of any fracture, hip fracture, non-vertebral fracture, or vertebral fracture. However, we found an excess risk of any fracture in men with SH when compared with women if the follow-up duration was <10.0 years and in studies with a high level of adjustment. Sex hormone levels are important for skeletal health in younger and middle-aged men. The reduced gonadotropin and sex hormone levels in men with SH may contribute to their observed increase in fracture risk [44]. Interestingly, we found that women with SH had an excess risk of hip fracture in studies with a low level of adjustment, which could be explained by the significant reduction in bone mineral density at the femoral neck in women with SH, which has not been observed in men [45]. However, our stratified analyses based on the level of adjustment could have affect the pooled effect estimates of the sex difference because the characteristics of the study participants could have influenced the risk of fracture.

This study has several shortcomings that should be acknowledged. First, it included both prospective and retrospective cohort studies, and the pooled conclusions could be biased by uncontrolled confounding factors. Second, the TSH level varied during follow-up, which could affect the risk of major cardiovascular outcomes and fractures. Third, the background treatments for STD were not adjusted for in most of the studies, which could have affected the risks of major cardiovascular outcomes and fractures related to STD. Fourth, the results of our analysis are based on both indirect and direct comparisons, and the heterogeneity in characteristics between men and women could have contributed to these risks. Finally, there are some inherent limitations to a meta-analysis based on published articles, including inevitable publication bias and restriction of analyses.

This study found no sex-related differences in the associations of STD with MACE and fractures. However, subgroup analysis indicated potential sex differences in the risks of MACE, any fracture, and hip fracture related to SH, suggesting that individuals at high risk should be carefully monitored. Further large-scale prospective cohort studies should be performed to confirm if there are sex differences in the association of STD with MACE and fractures.

## Supplementary Materials

Supplementary Figures

Supplementary Table 1
