# Supplementary material for: Sex differences in major cardiovascular outcomes and fractures in patients with subclinical thyroid dysfunction: a systematic review and meta-analysis
Source: Aging (Albany NY). 2022 Oct 25;14(20):8448–85. doi: 10.18632/aging.204352 (PMC9648794; doi:10.18632/aging.204352)
Supplement: Supplementary Figures [file aging-14-204352-s001.pdf]

SUPPLEMENTARY FIGURES

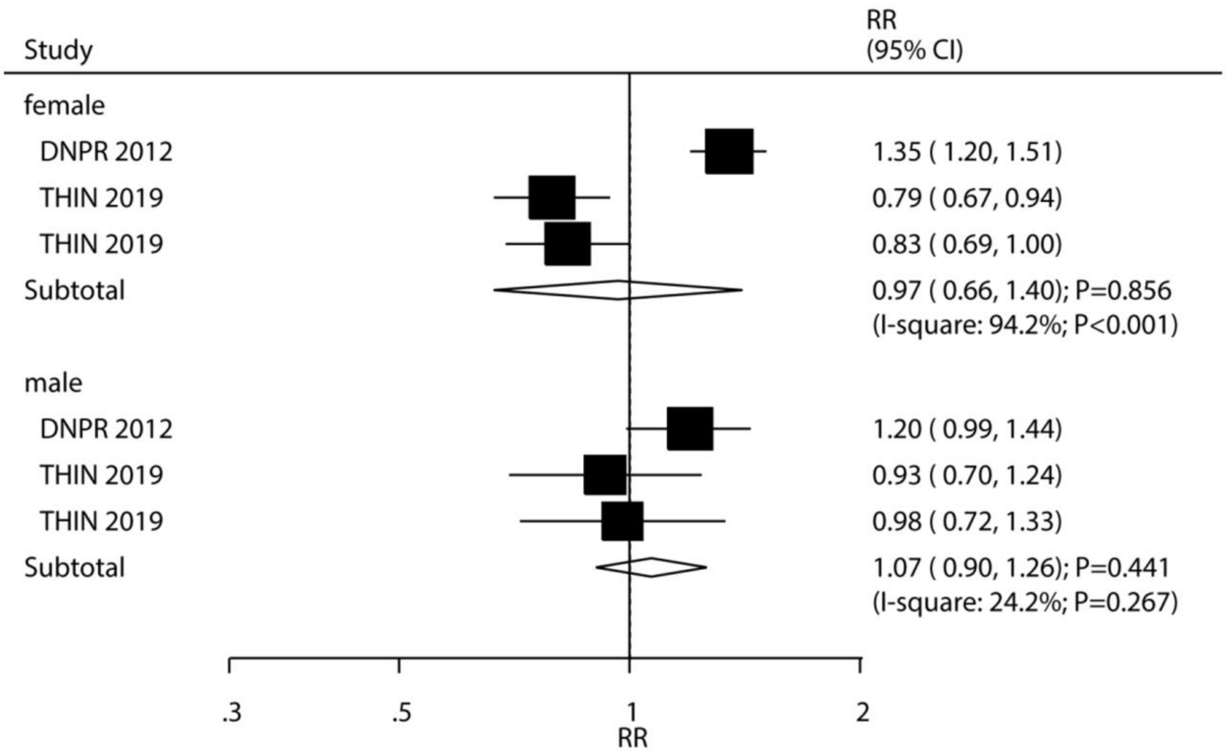

Supplementary Figure 1. Association of subclinical hyperthyroidism with the risk of atrial fibrillation.

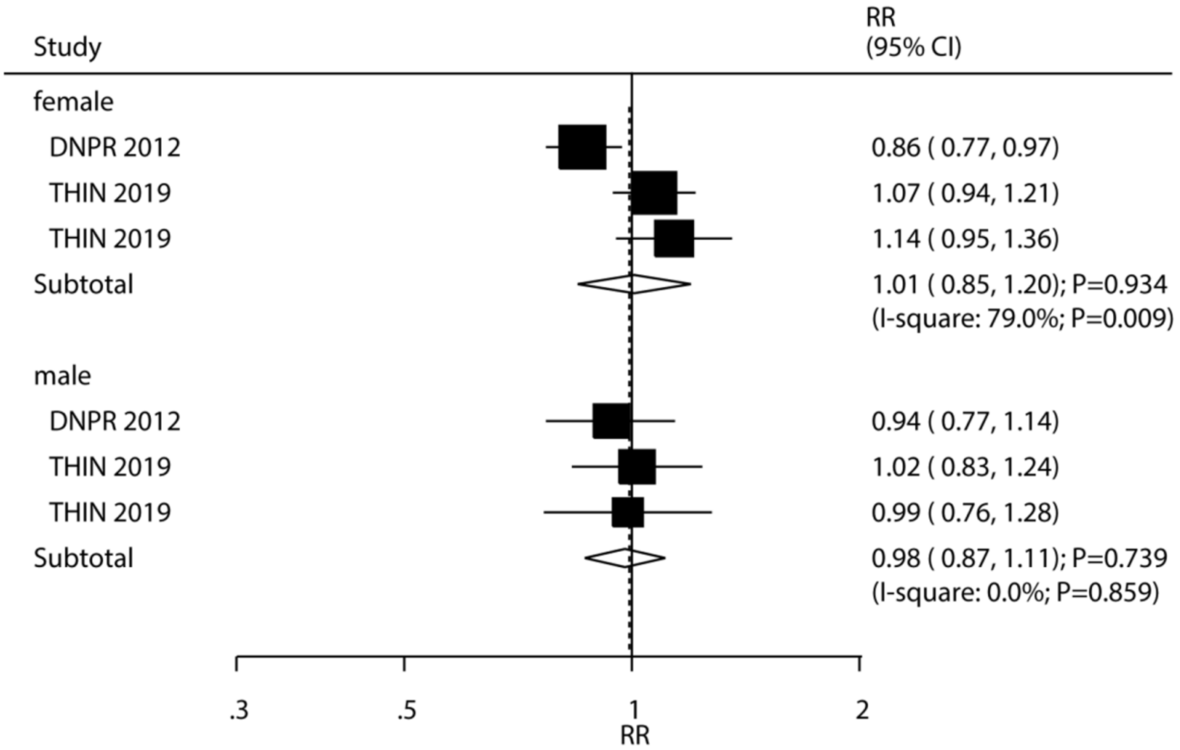

Supplementary Figure 2. Association of subclinical hypothyroidism with the risk of atrial fibrillation.

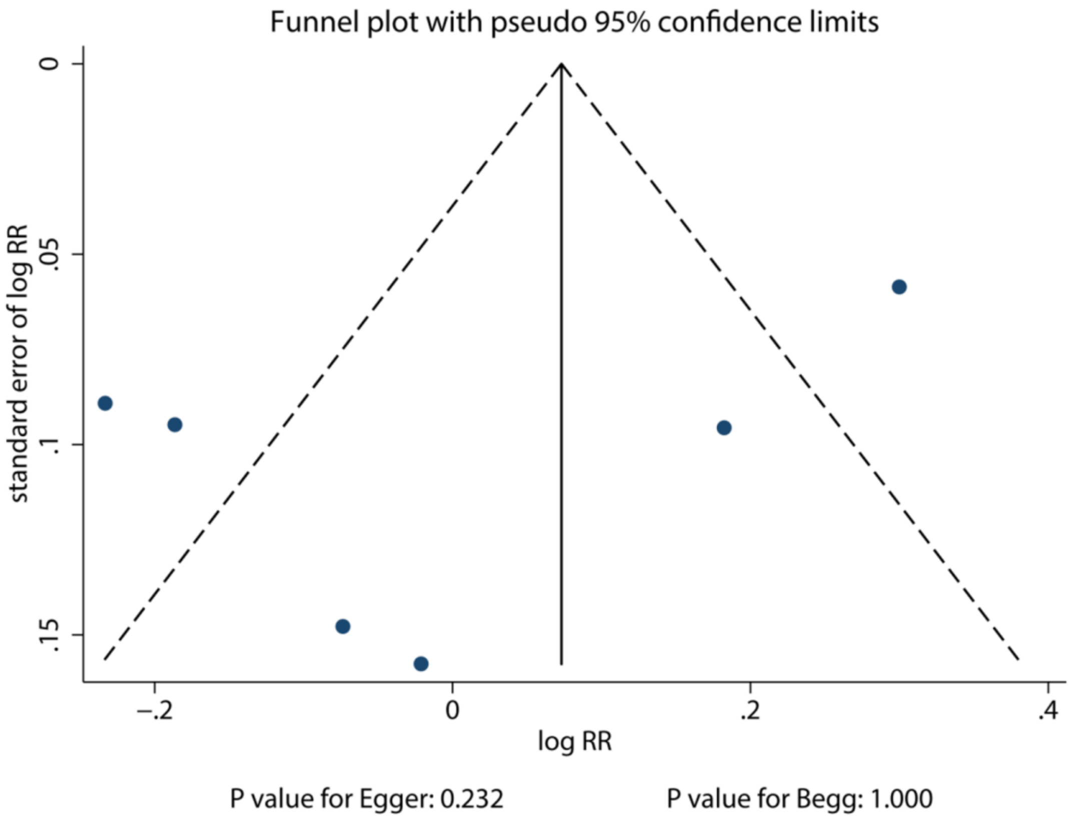

Supplementary Figure 3. Funnel plot for the association of subclinical hyperthyroidism with the risk of atrial fibrillation.

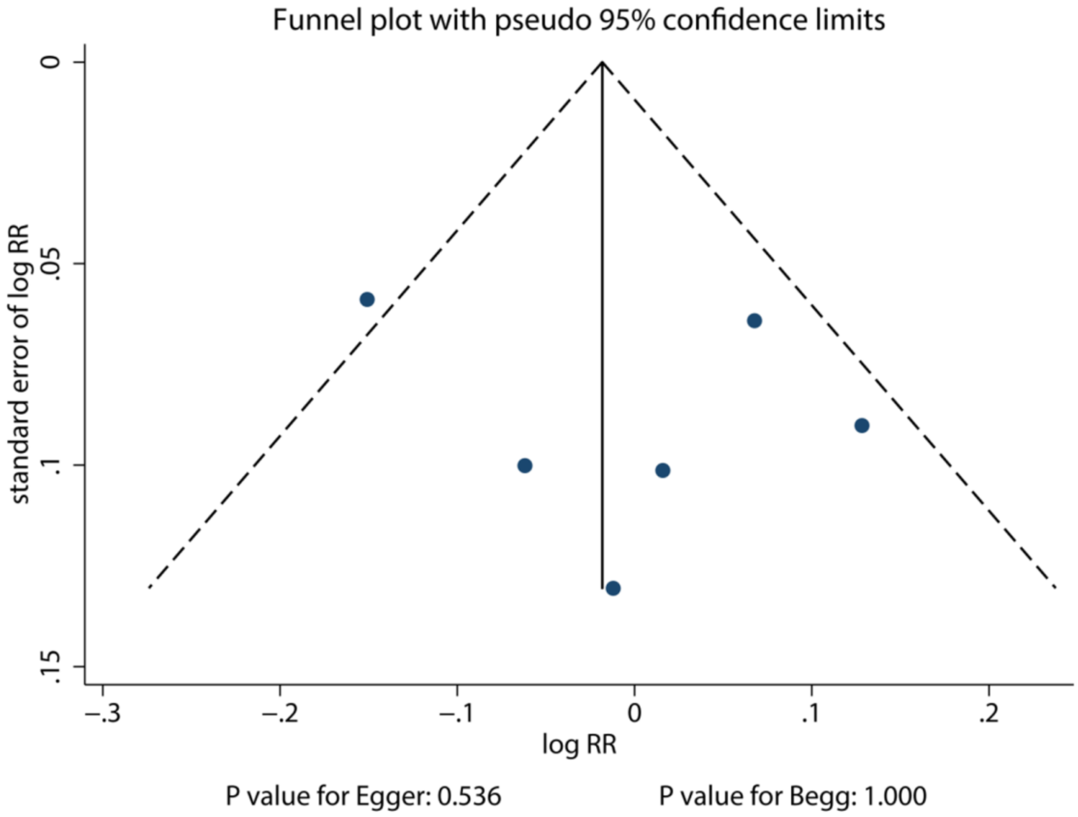

Supplementary Figure 4. Funnel plot for the association of subclinical hypothyroidism with the risk of atrial fibrillation.

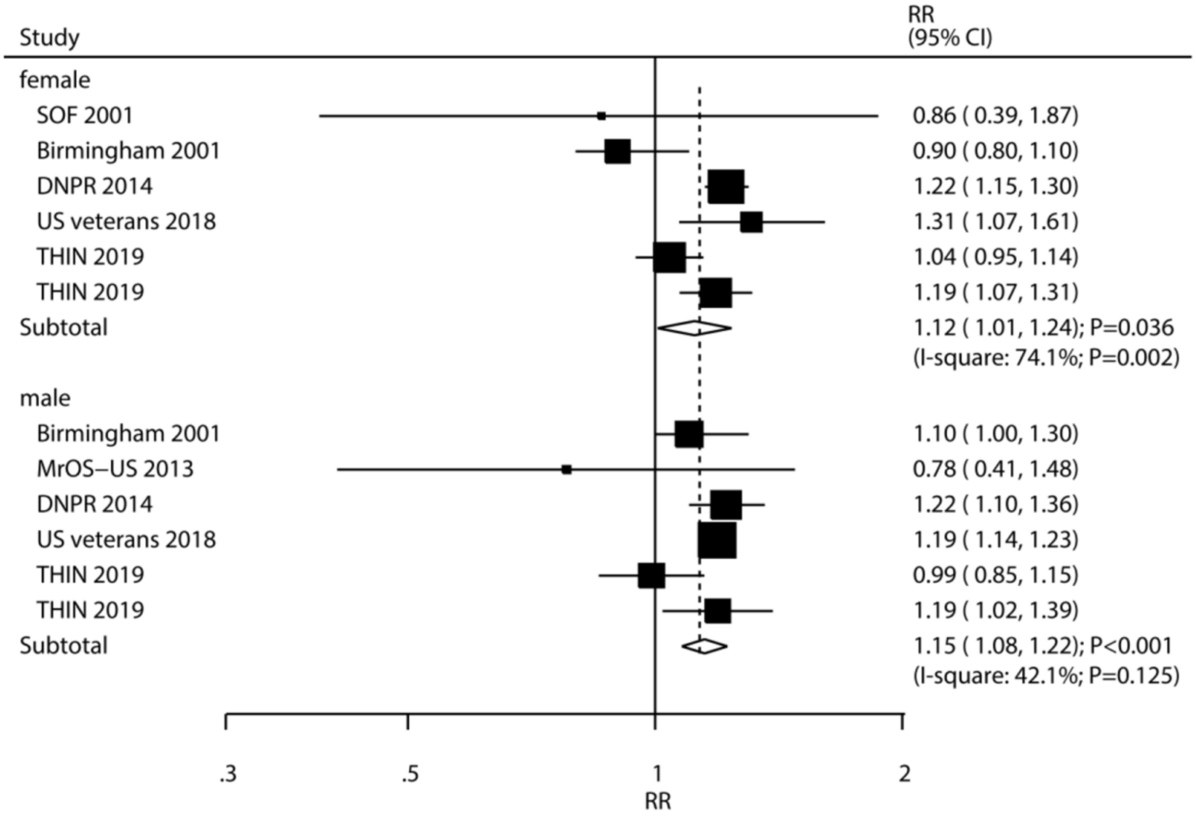

Supplementary Figure 5. Association of subclinical hyperthyroidism with the risk of all-cause mortality.

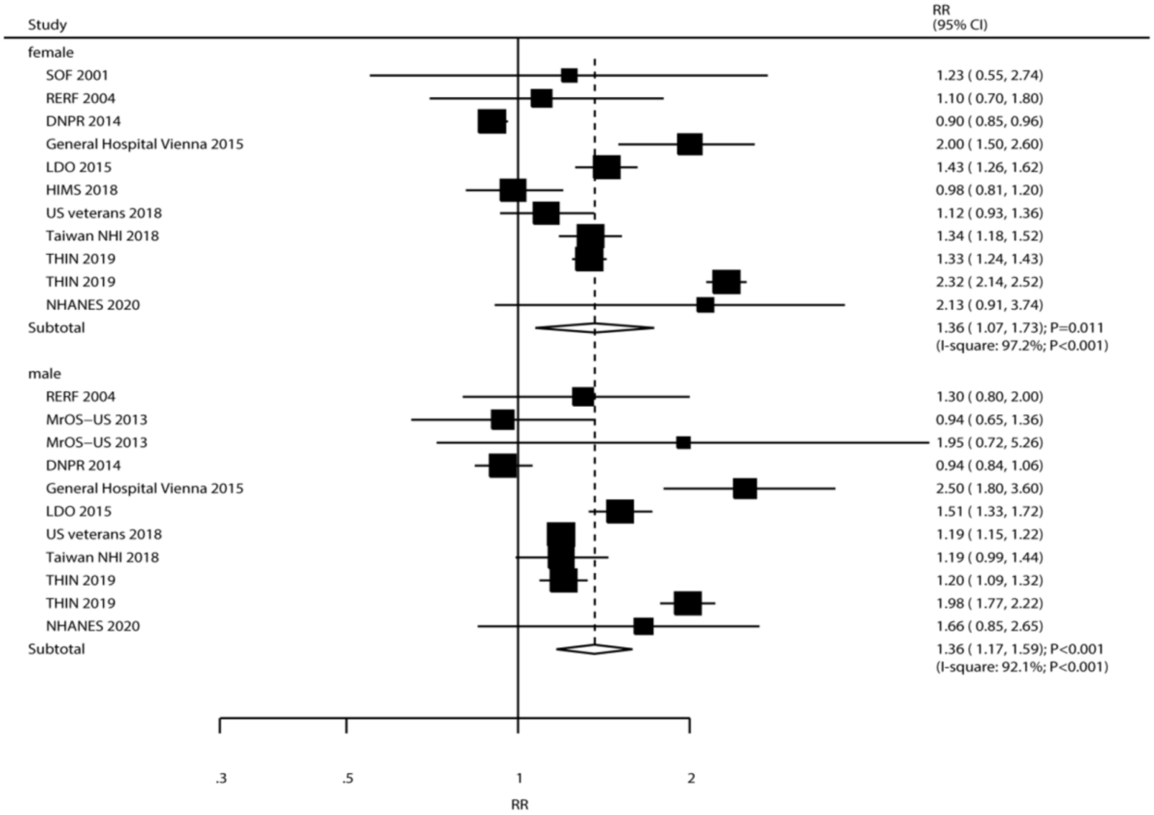

Supplementary Figure 6. Association of subclinical hypothyroidism with the risk of all-cause mortality.

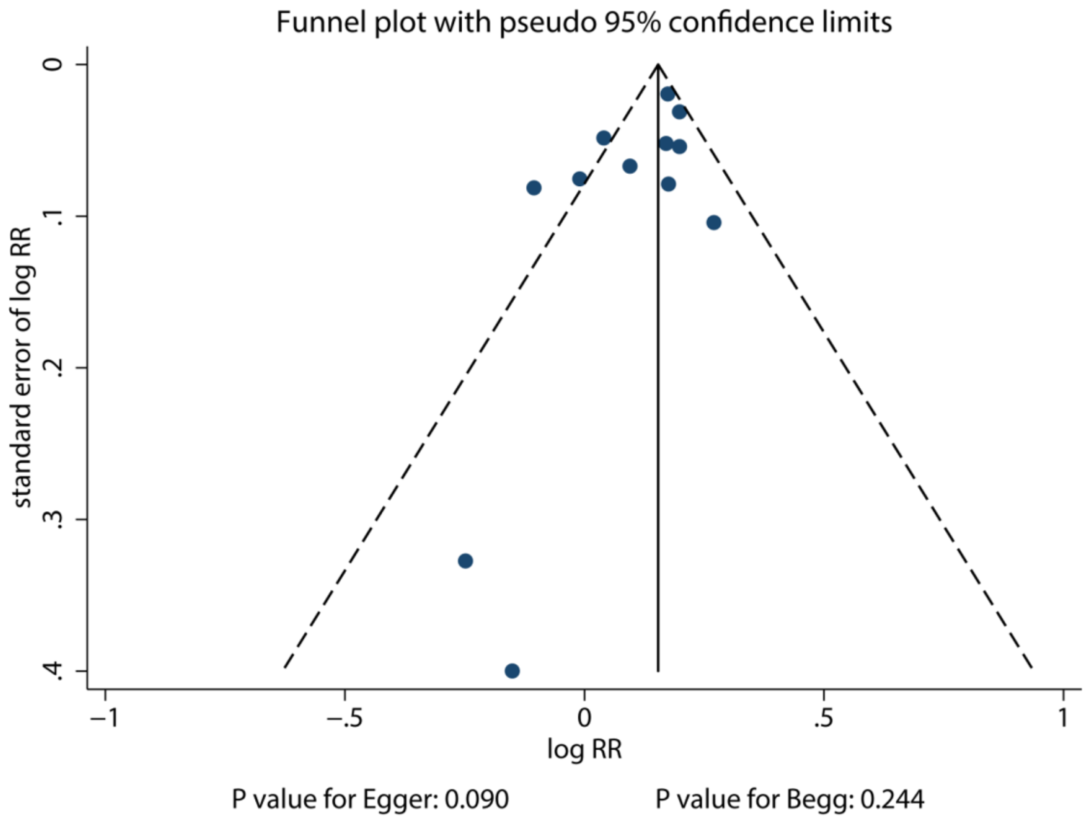

Supplementary Figure 7. Funnel plot for the association of subclinical hyperthyroidism with the risk of all-cause mortality.

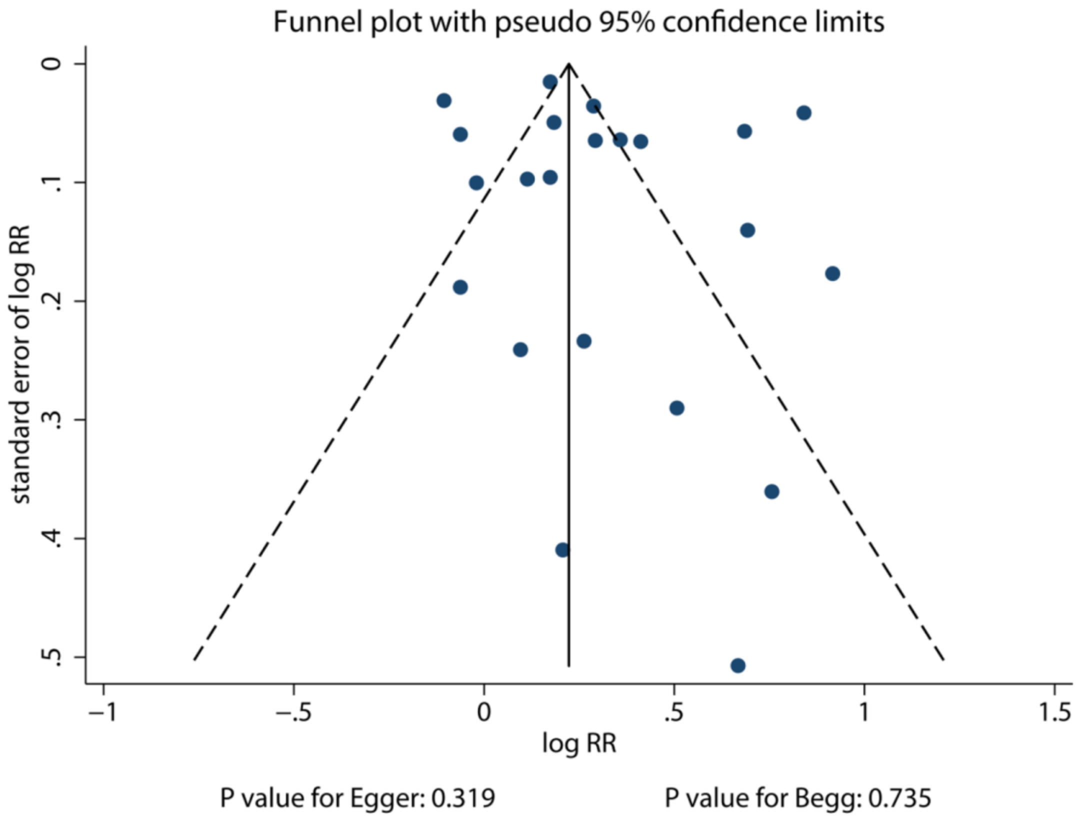

Supplementary Figure 8. Funnel plot for the association of subclinical hypothyroidism with the risk of all-cause mortality.

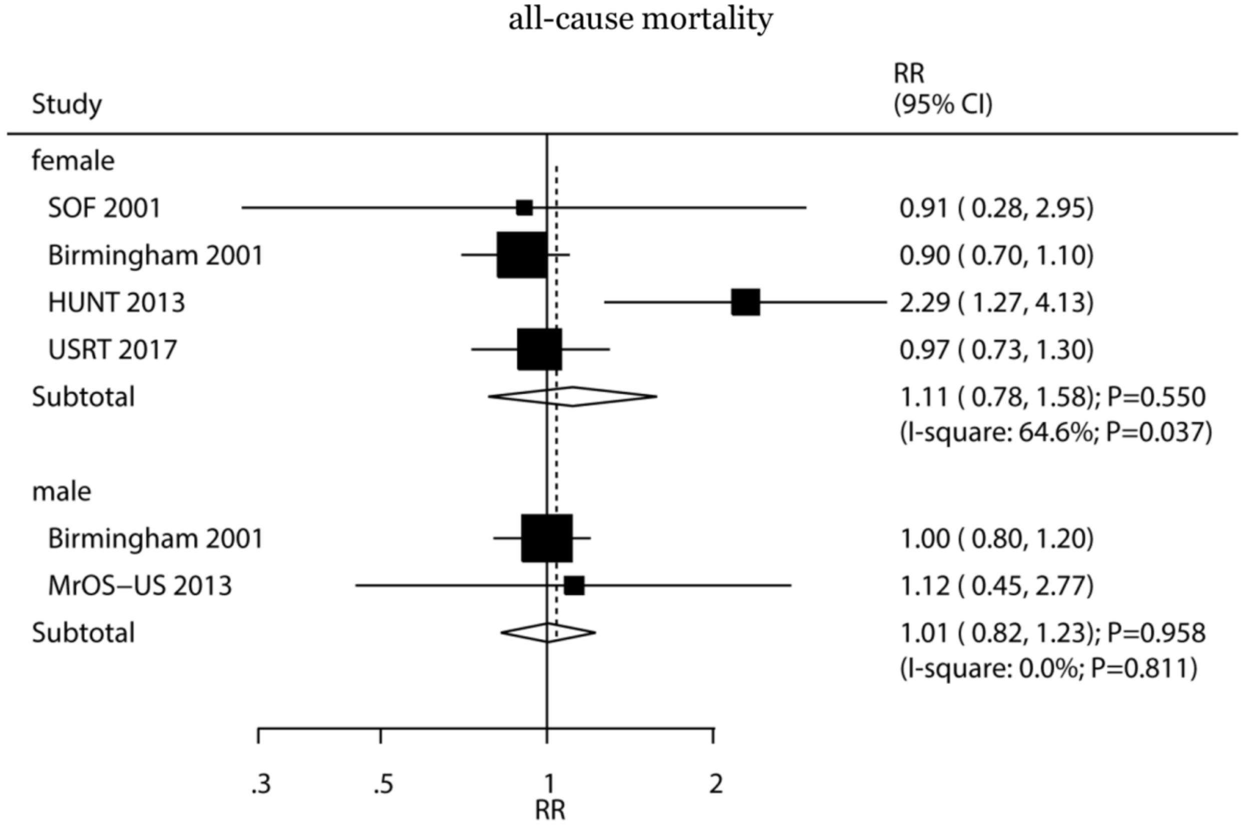

Supplementary Figure 9. Association of subclinical hyperthyroidism with the risk of cardiac death.

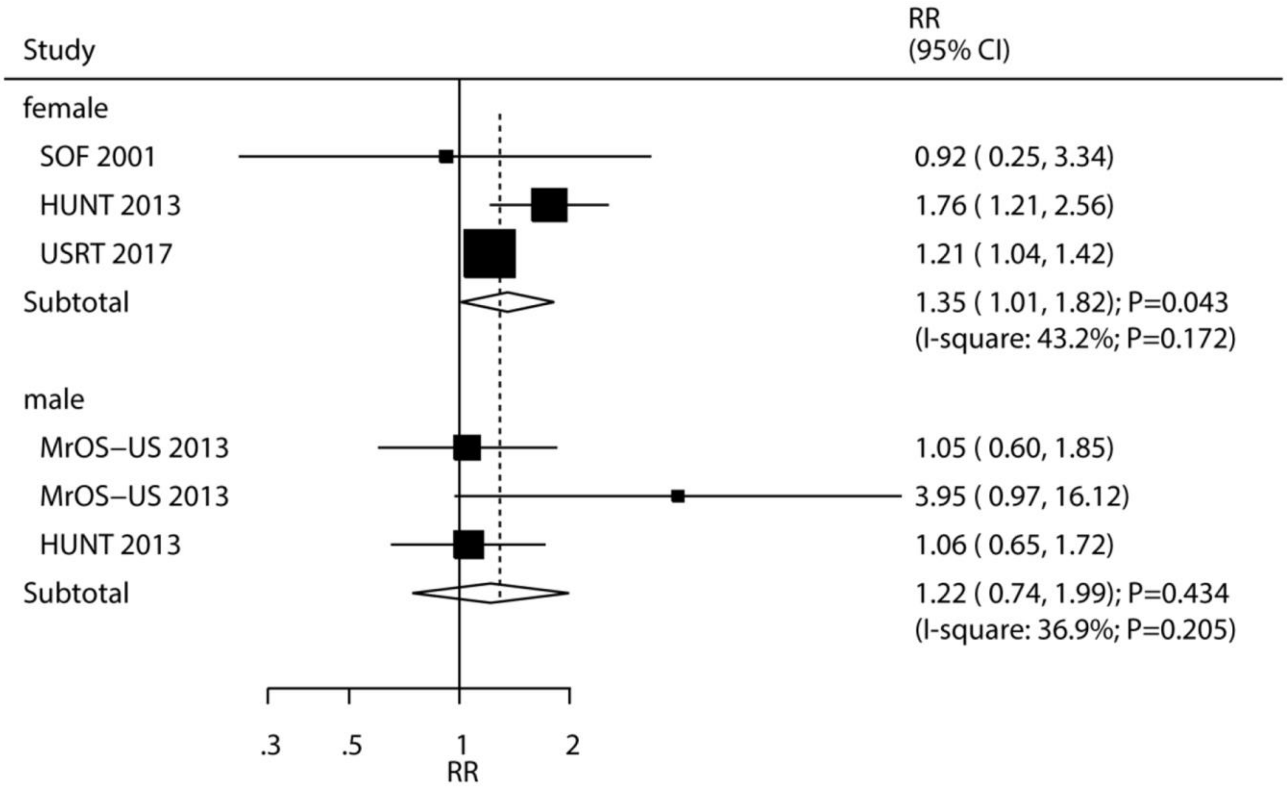

Supplementary Figure 10. Association of subclinical hypothyroidism with the risk of cardiac death.

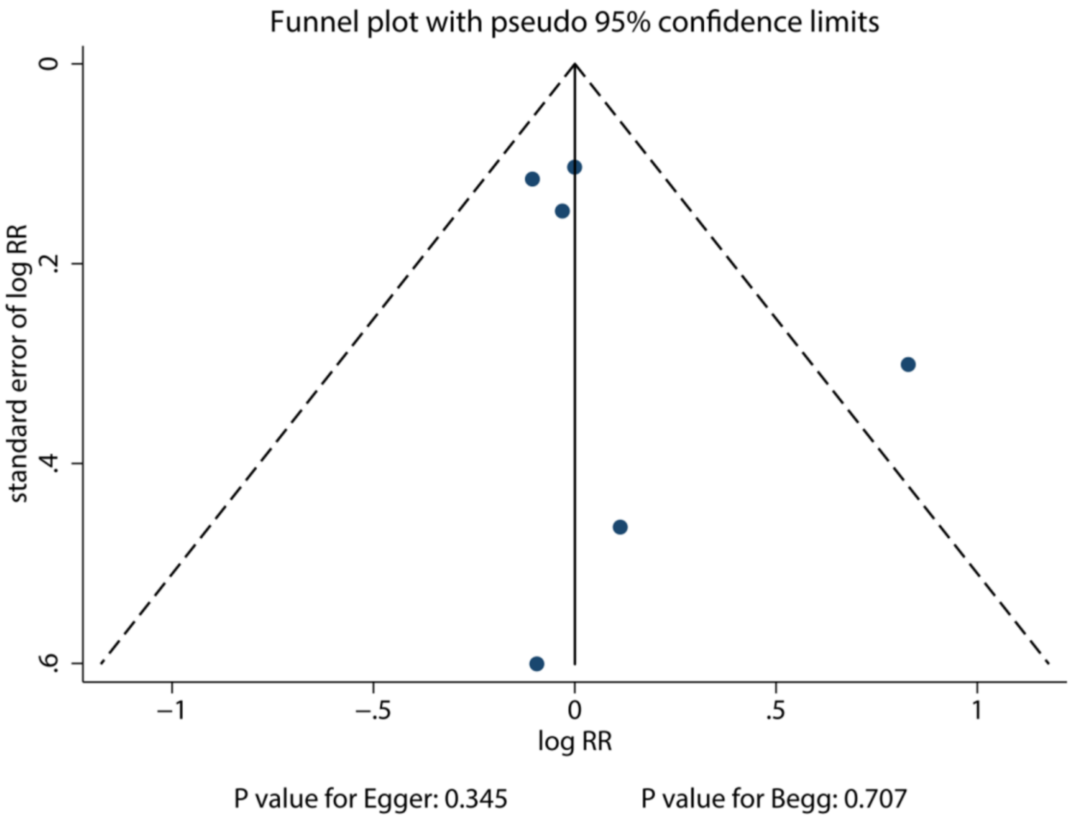

Supplementary Figure 11. Funnel plot for the association of subclinical hyperthyroidism with the risk of cardiac death.

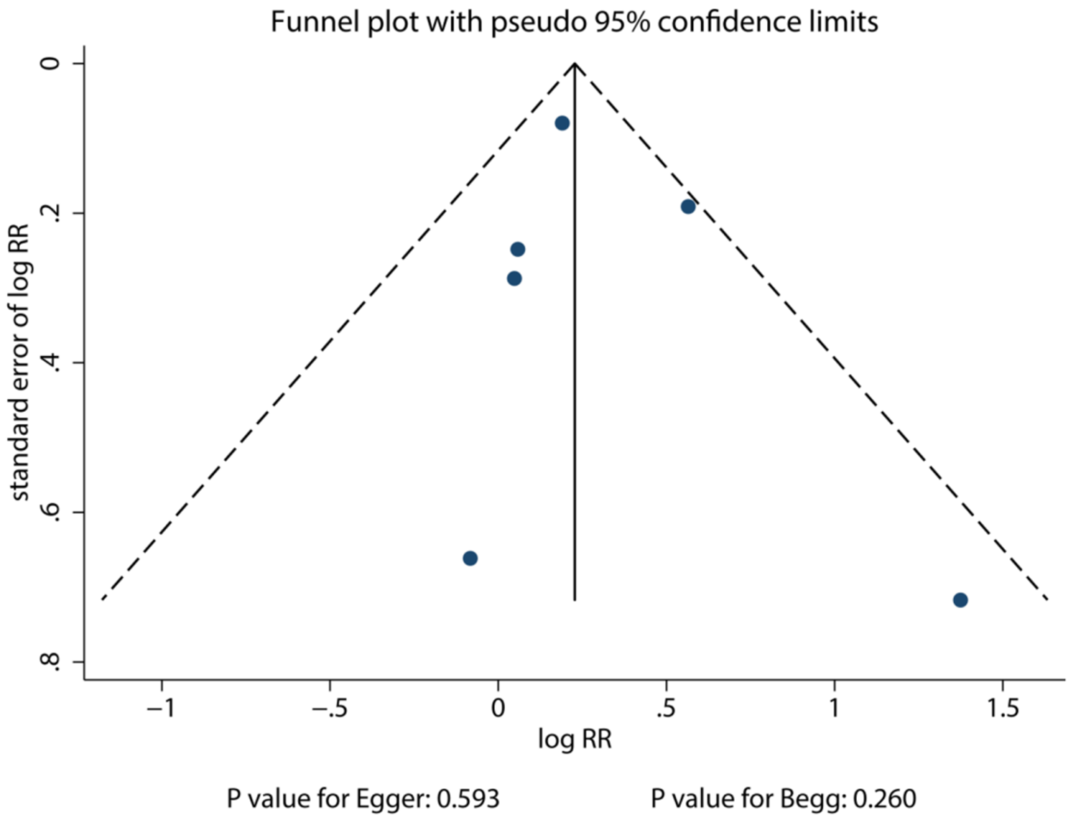

Supplementary Figure 12. Funnel plot for the association of subclinical hypothyroidism with the risk of cardiac death.

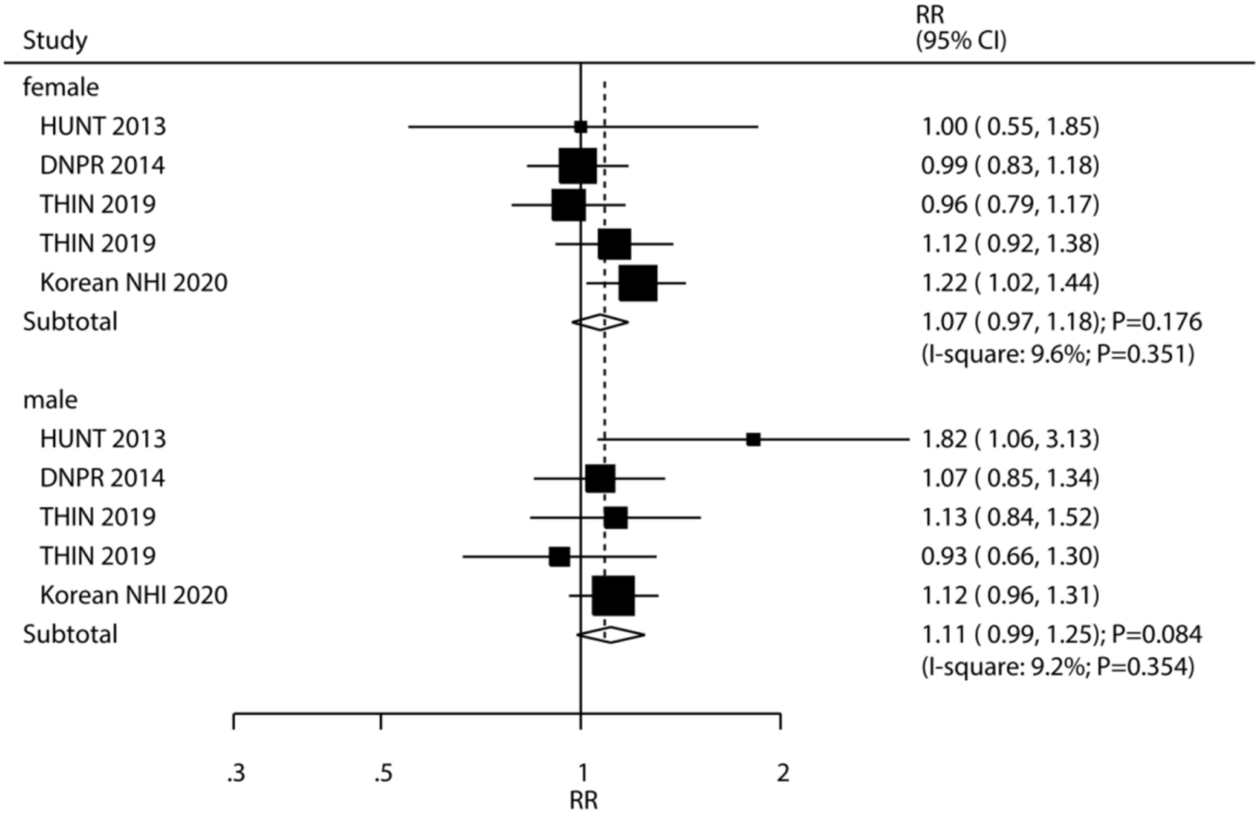

Supplementary Figure 13. Association of subclinical hyperthyroidism with the risk of coronary heart disease.

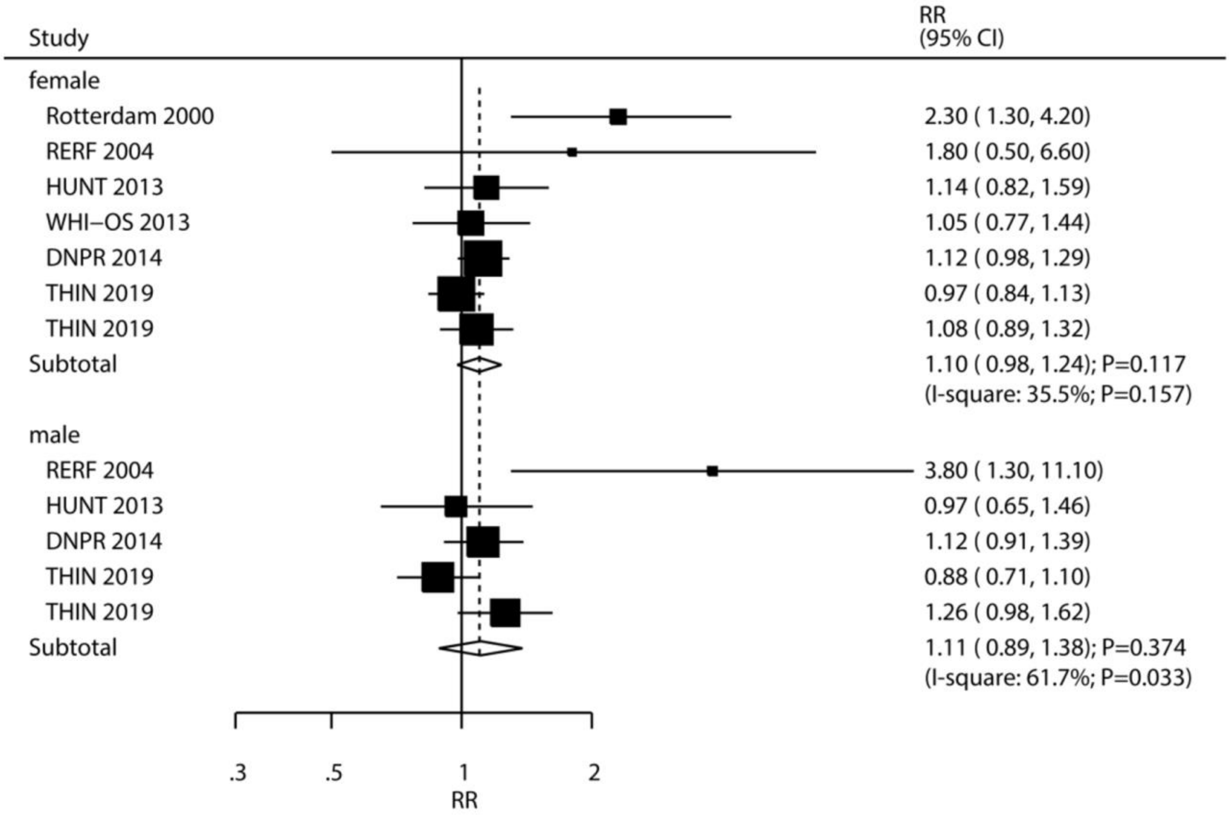

Supplementary Figure 14. Association of subclinical hypothyroidism with the risk of coronary heart disease.

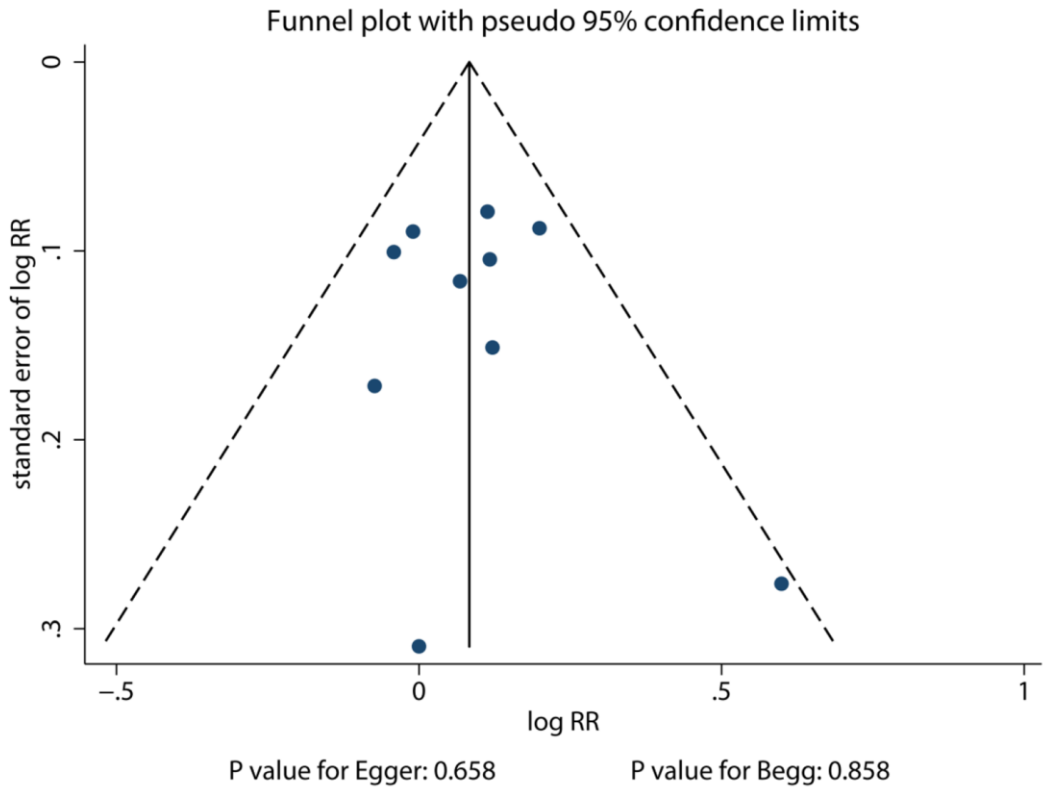

Supplementary Figure 15. Funnel plot for the association of subclinical hyperthyroidism with the risk of coronary heart disease.

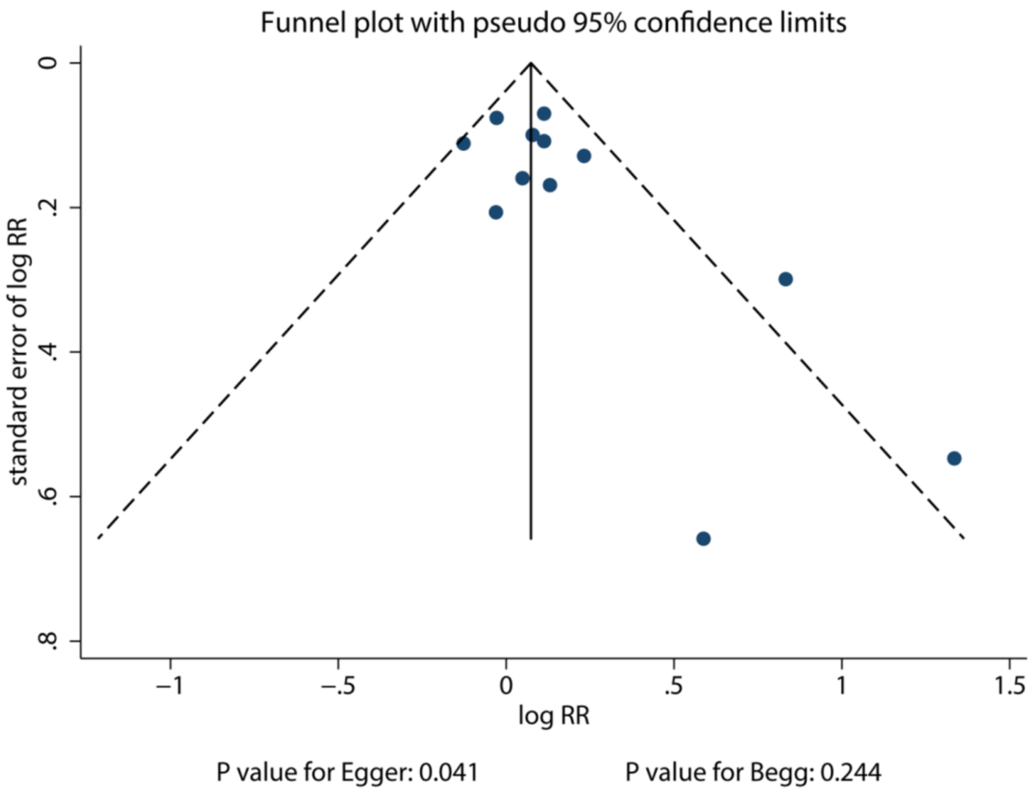

Supplementary Figure 16. Funnel plot for the association of subclinical hypothyroidism with the risk of coronary heart disease.

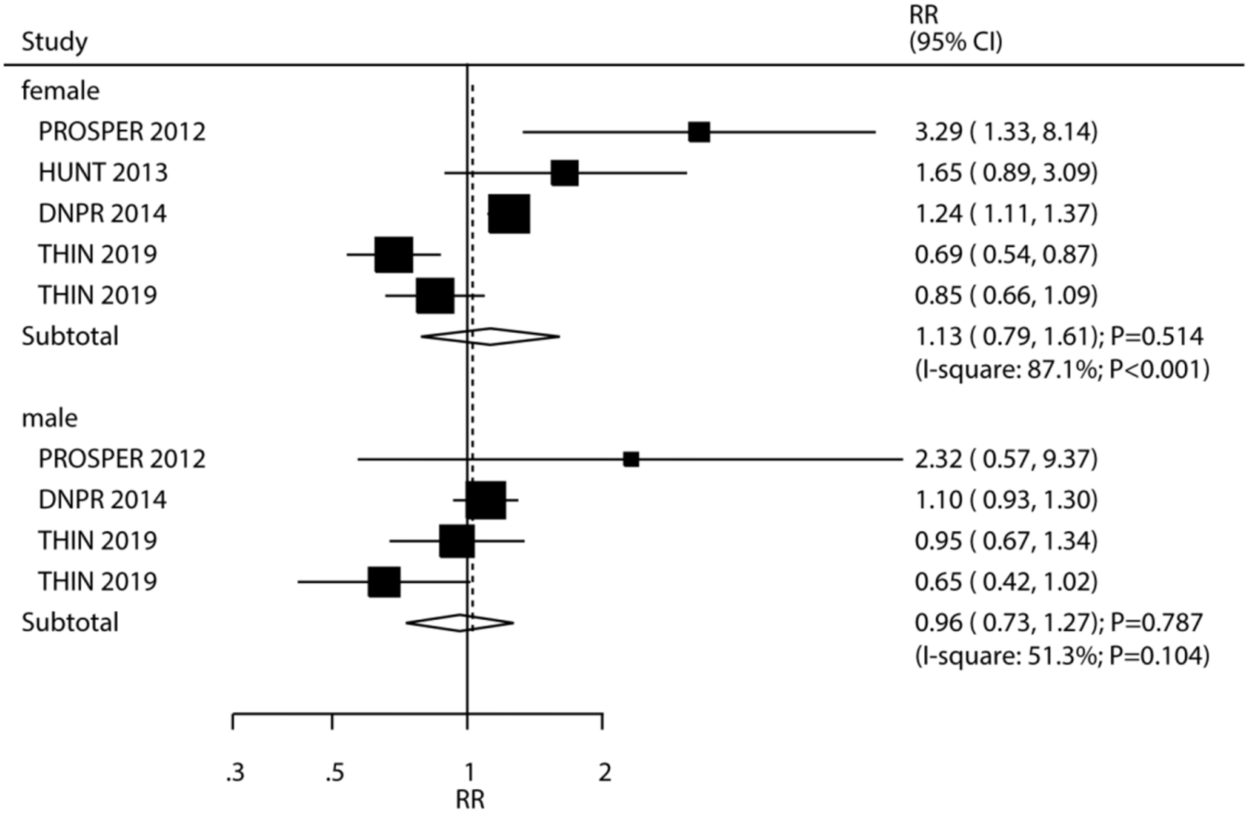

Supplementary Figure 17. Association of subclinical hyperthyroidism with the risk of heart failure.

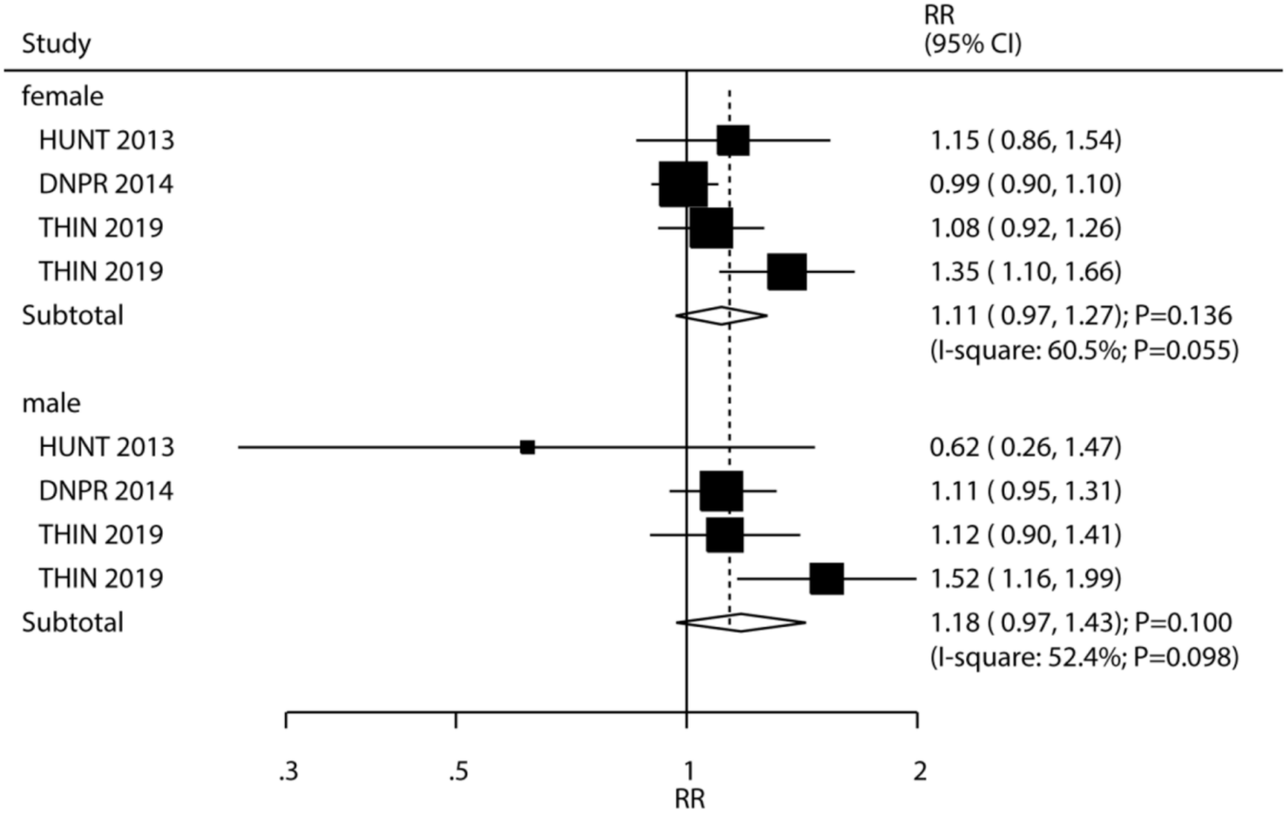

Supplementary Figure 18. Association of subclinical hypothyroidism with the risk of heart failure.

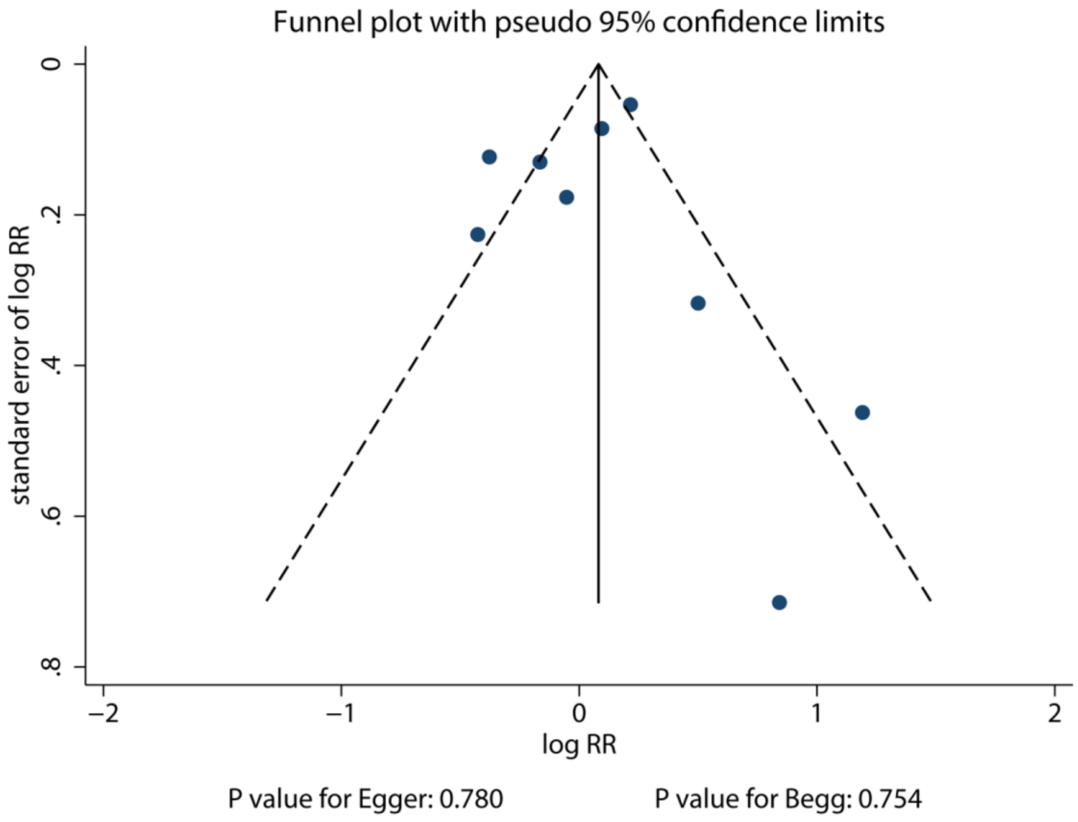

Supplementary Figure 19. Funnel plot for the association of subclinical hyperthyroidism with the risk of heart failure.

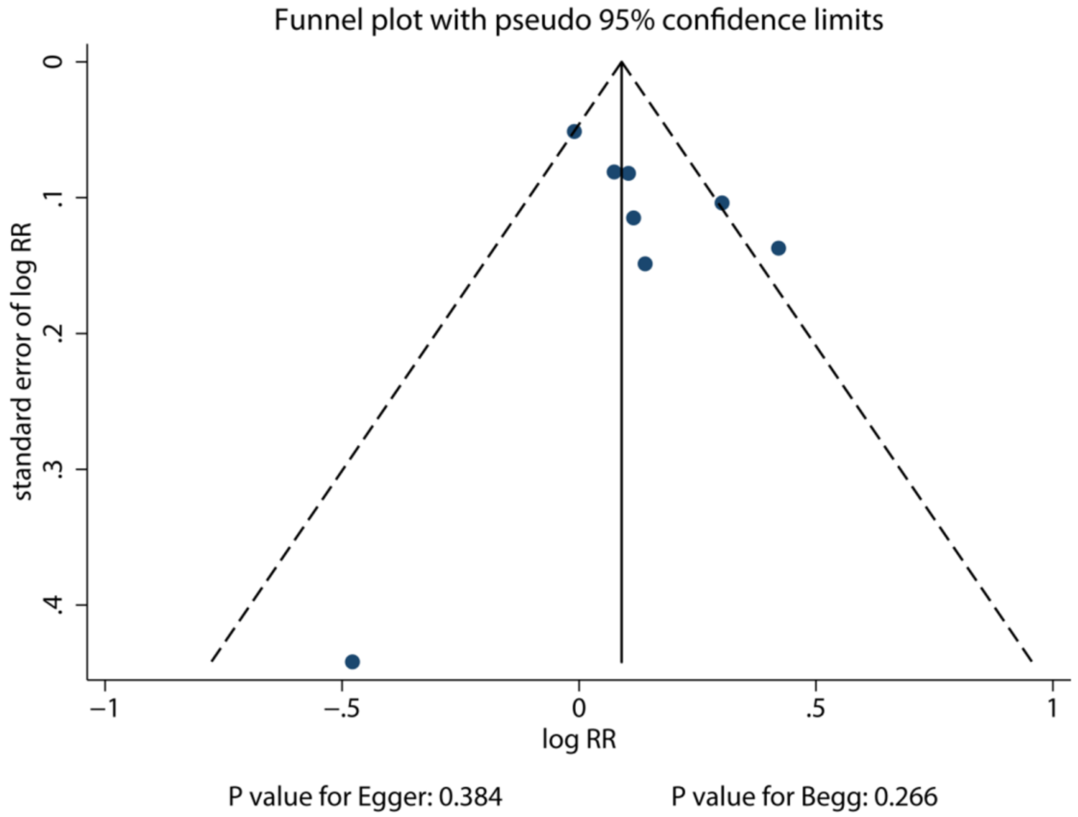

Supplementary Figure 20. Funnel plot for the association of subclinical hypothyroidism with the risk of heart failure.

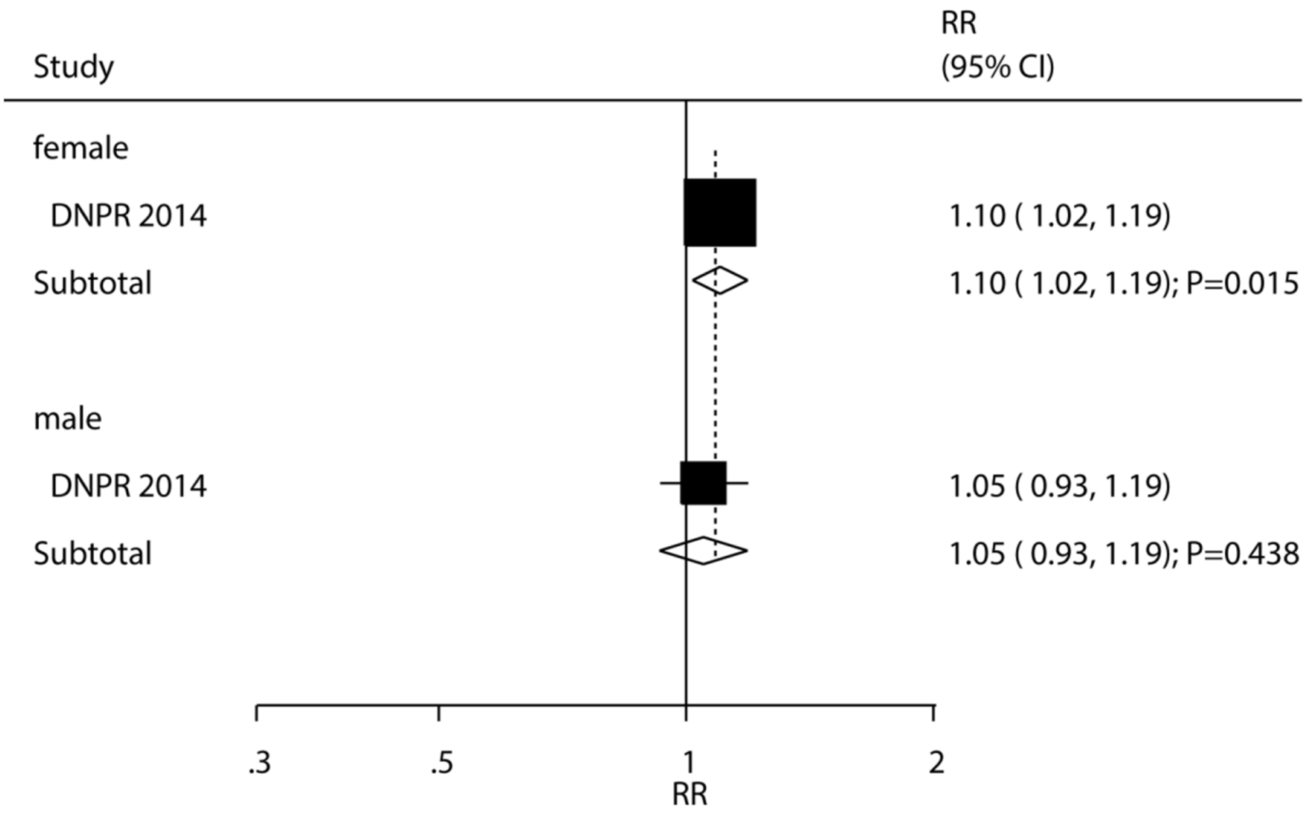

Supplementary Figure 21. Association of subclinical hyperthyroidism with the risk of major adverse cardiovascular events.

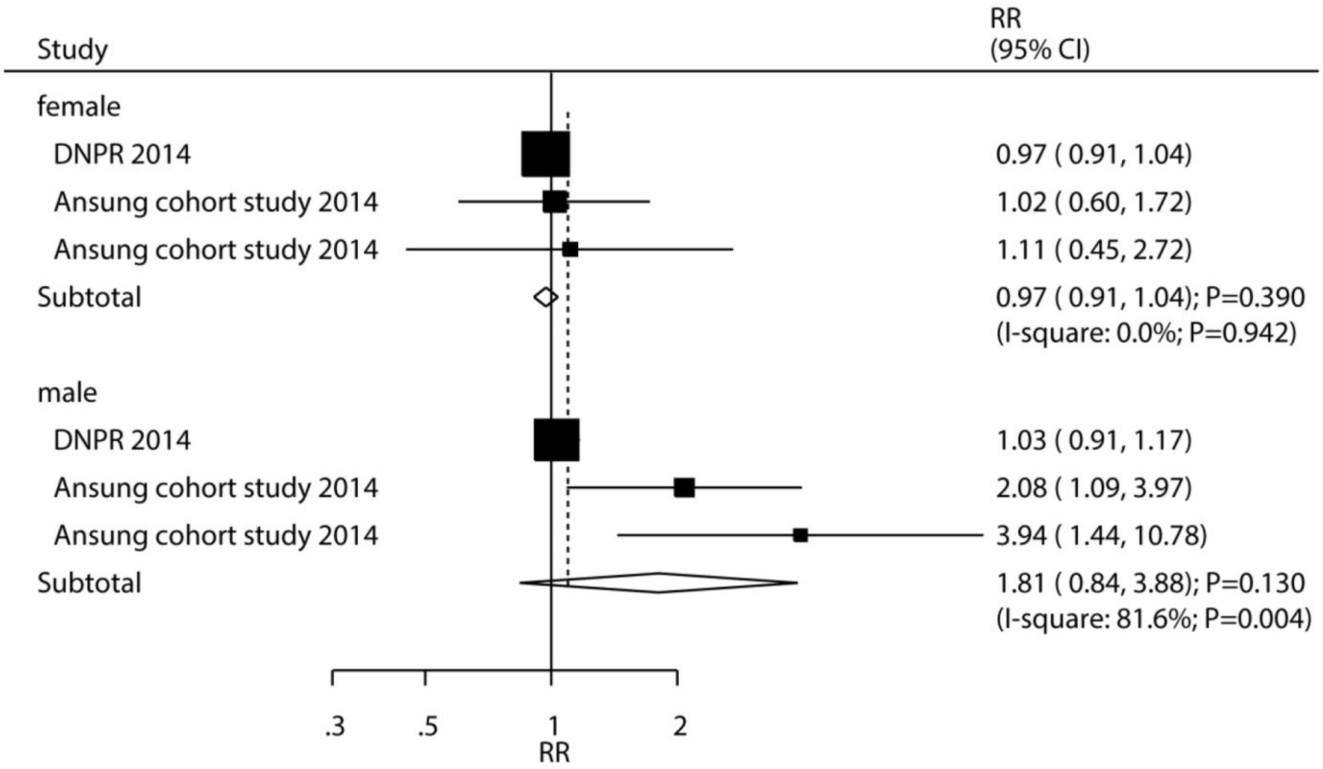

Supplementary Figure 22. Association of subclinical hypothyroidism with the risk of major adverse cardiovascular events.

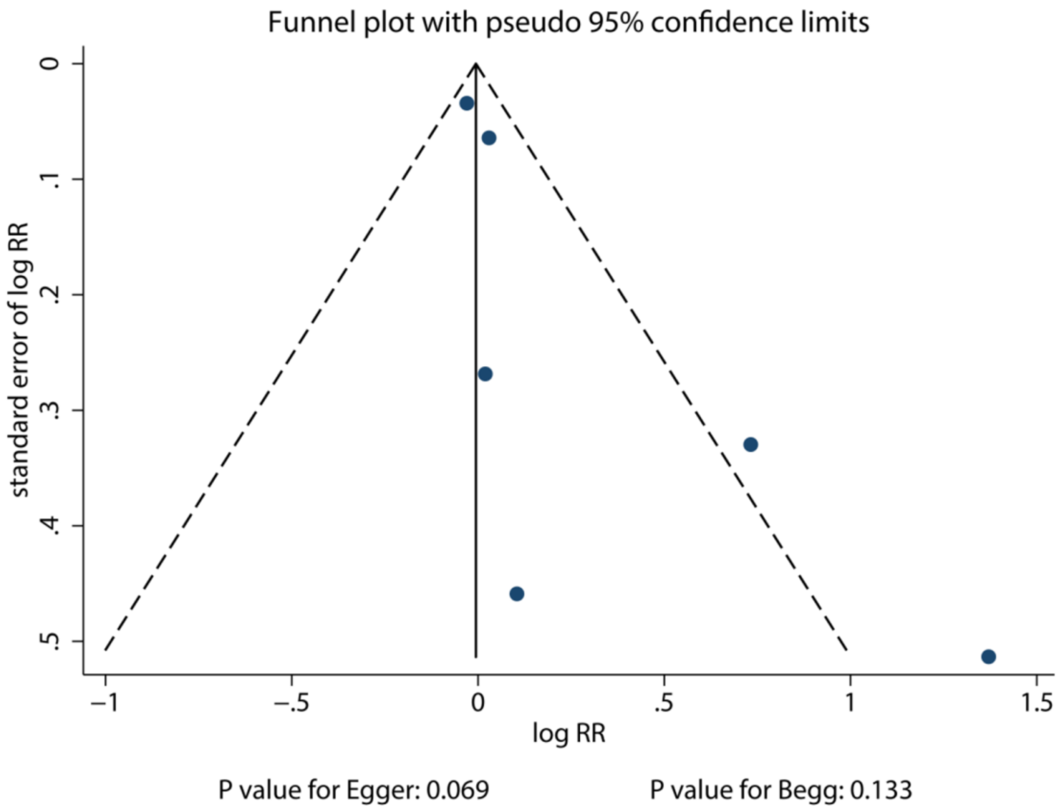

Supplementary Figure 23. Funnel plot for the association of subclinical hypothyroidism with the risk of major adverse cardiovascular events.

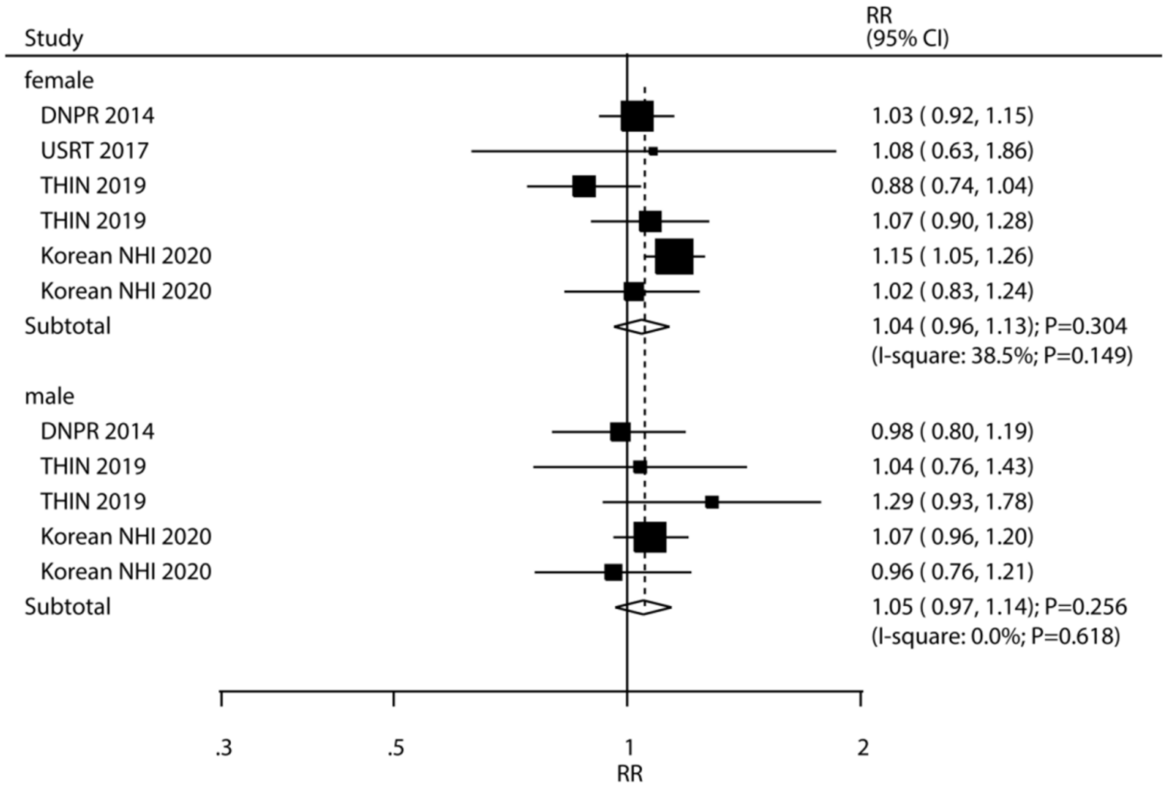

Supplementary Figure 24. Association of subclinical hyperthyroidism with the risk of stroke.

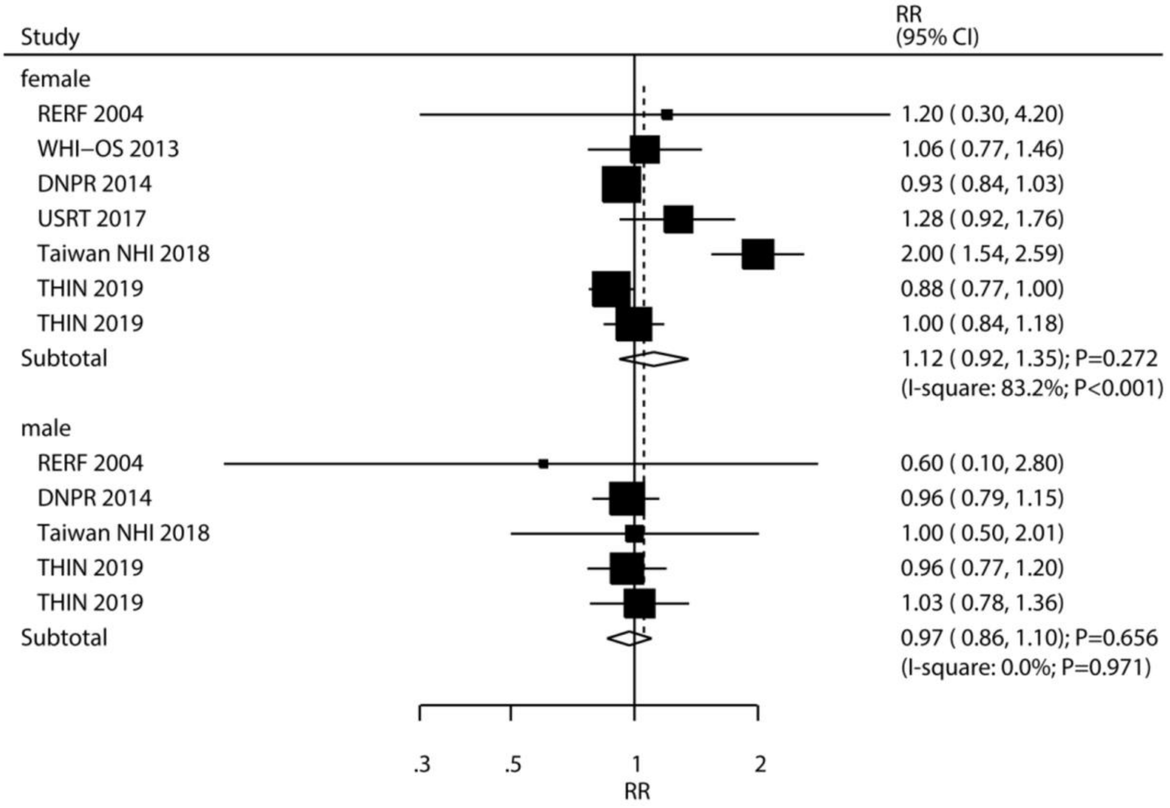

Supplementary Figure 25. Association of subclinical hypothyroidism with the risk of stroke.

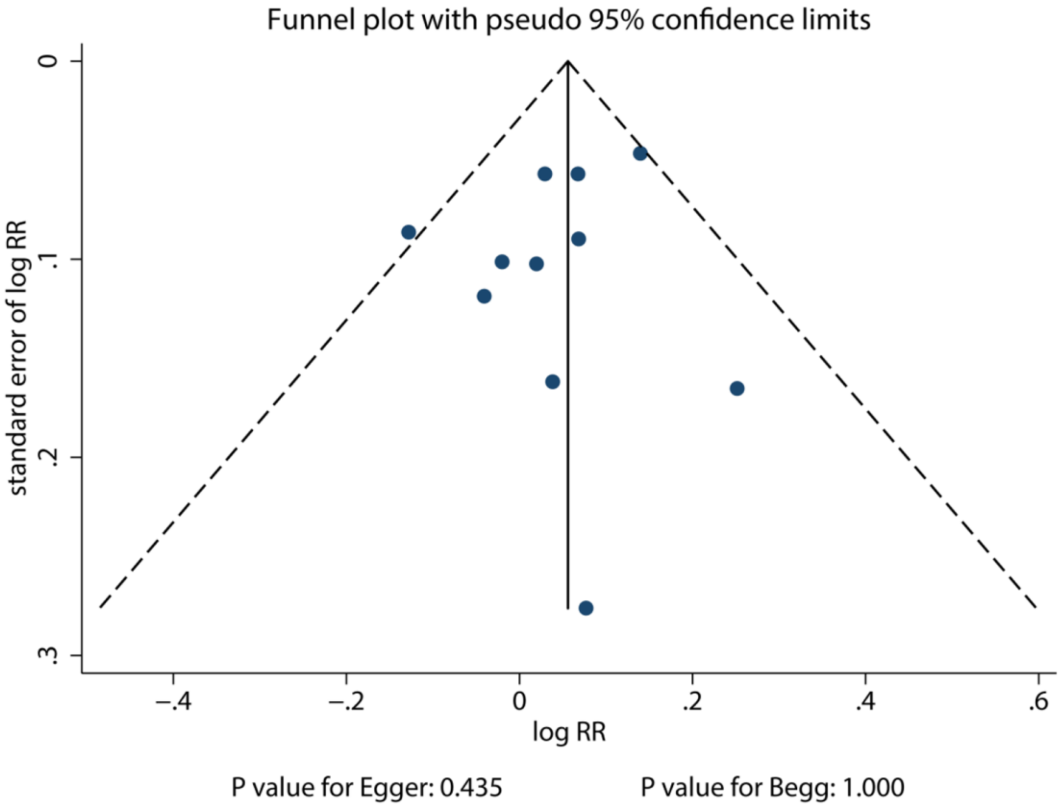

Supplementary Figure 26. Funnel plot for the association of subclinical hyperthyroidism with the risk of stroke.

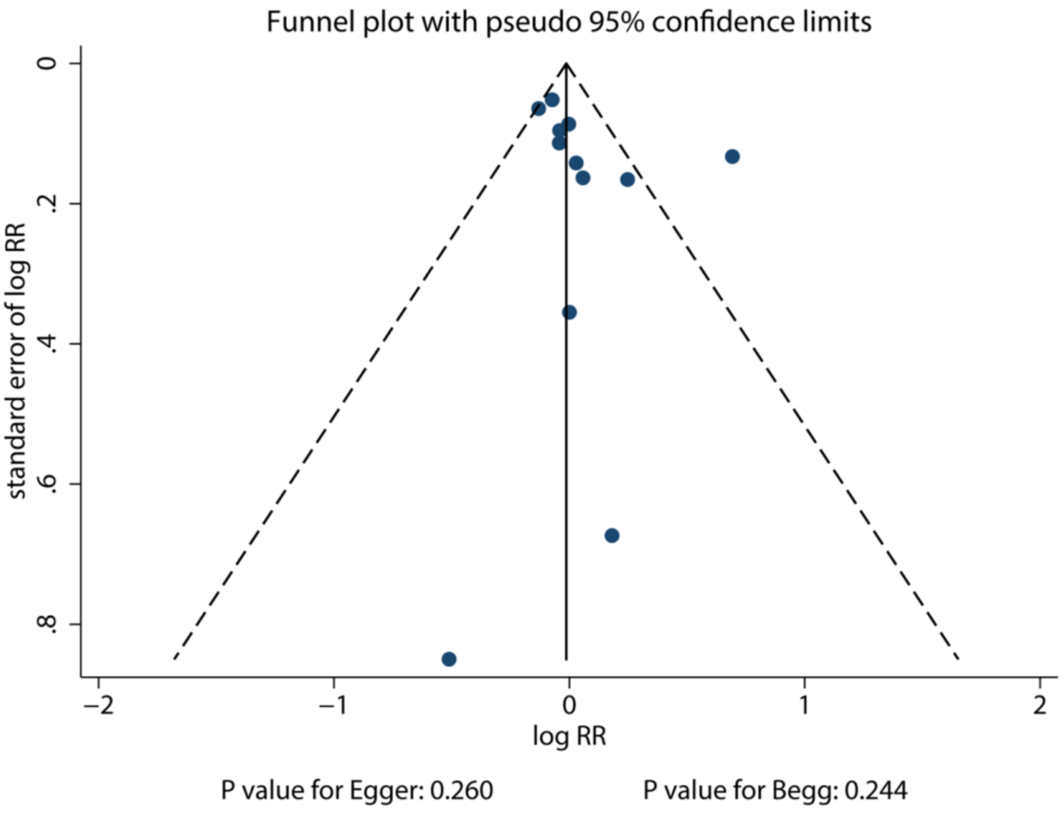

Supplementary Figure 27. Funnel plot for the association of subclinical hypothyroidism with the risk of stroke.

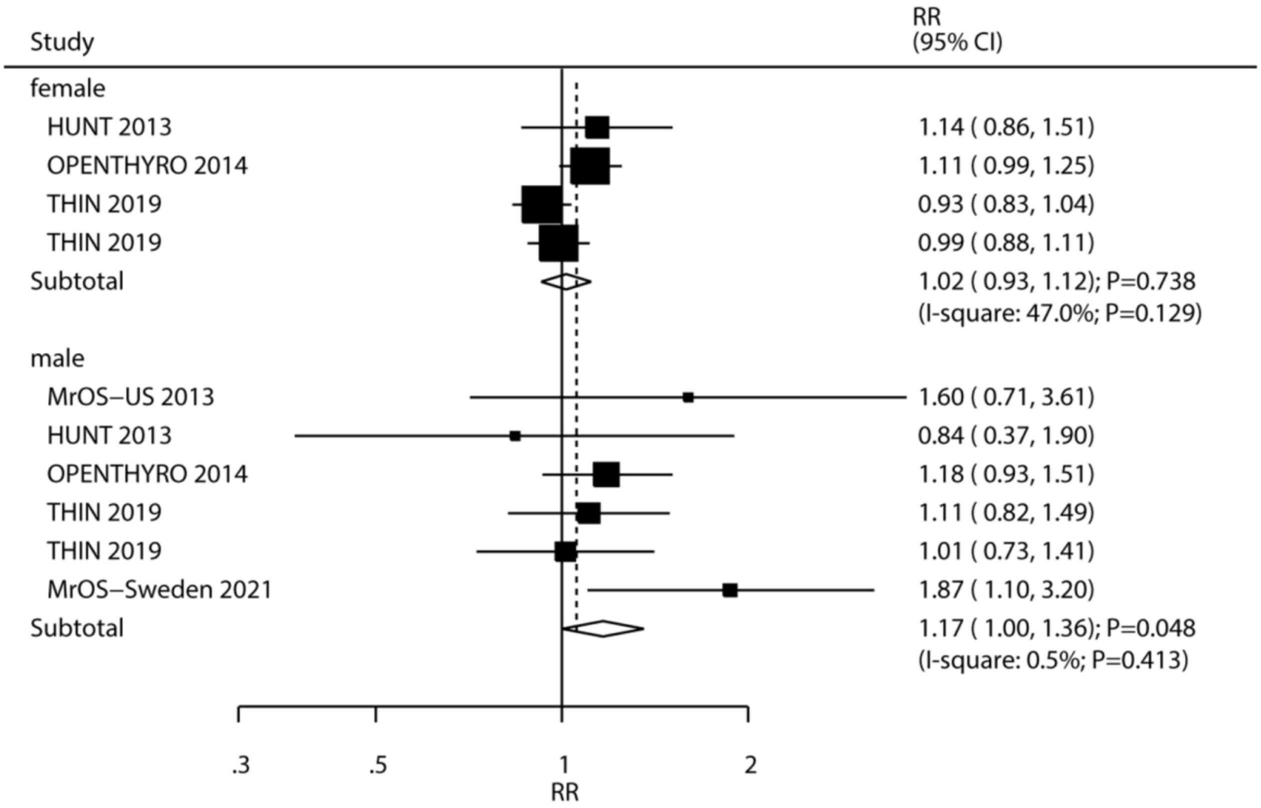

Supplementary Figure 28. Association of subclinical hyperthyroidism with the risk of any fracture.

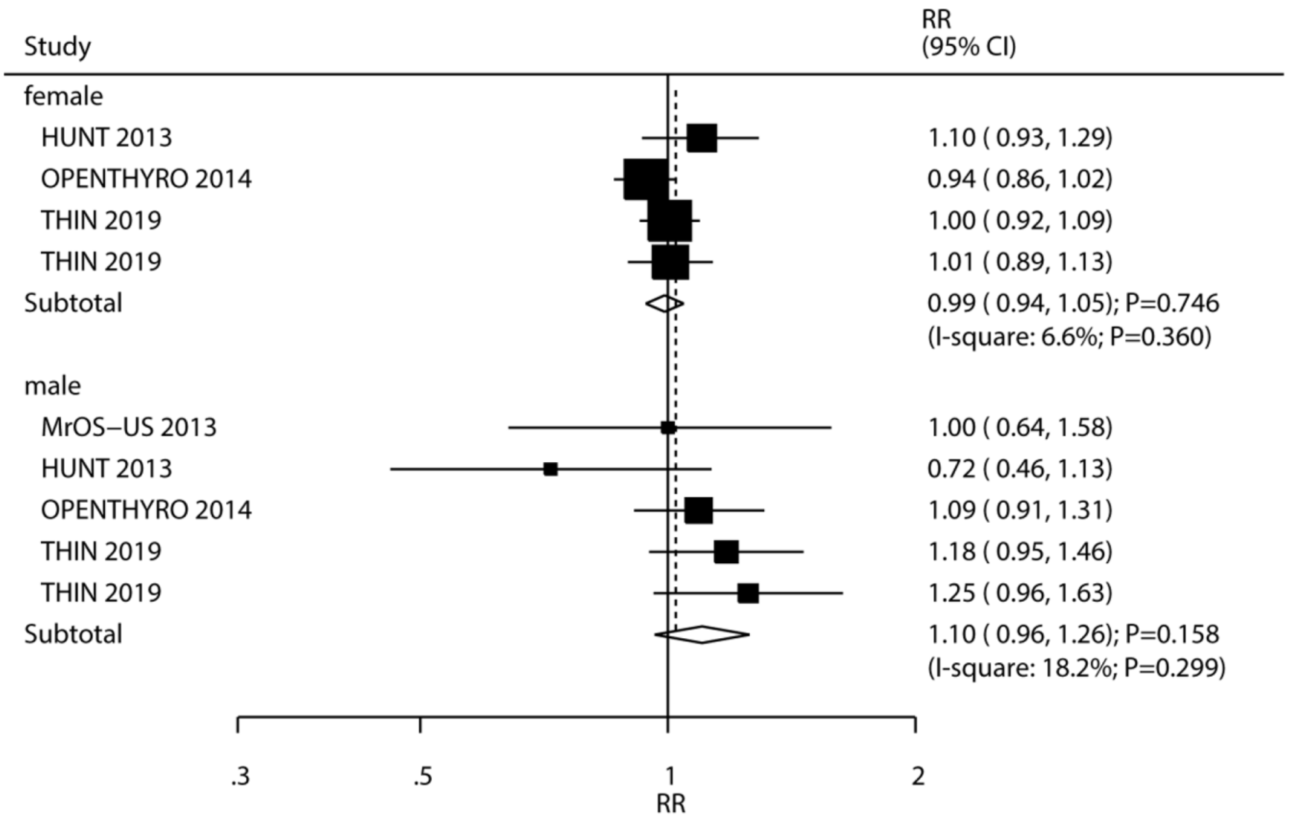

Supplementary Figure 29. Association of subclinical hypothyroidism with the risk of any fracture.

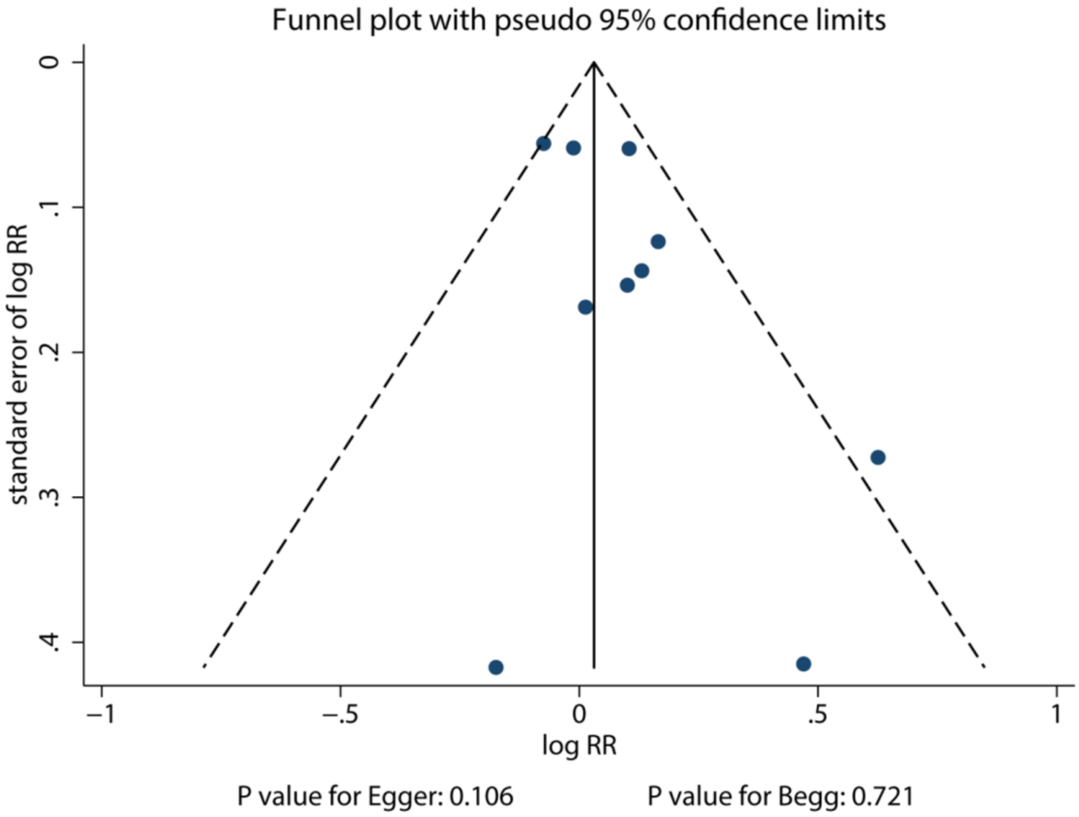

Supplementary Figure 30. Funnel plot for the association of subclinical hyperthyroidism with the risk of any fracture.

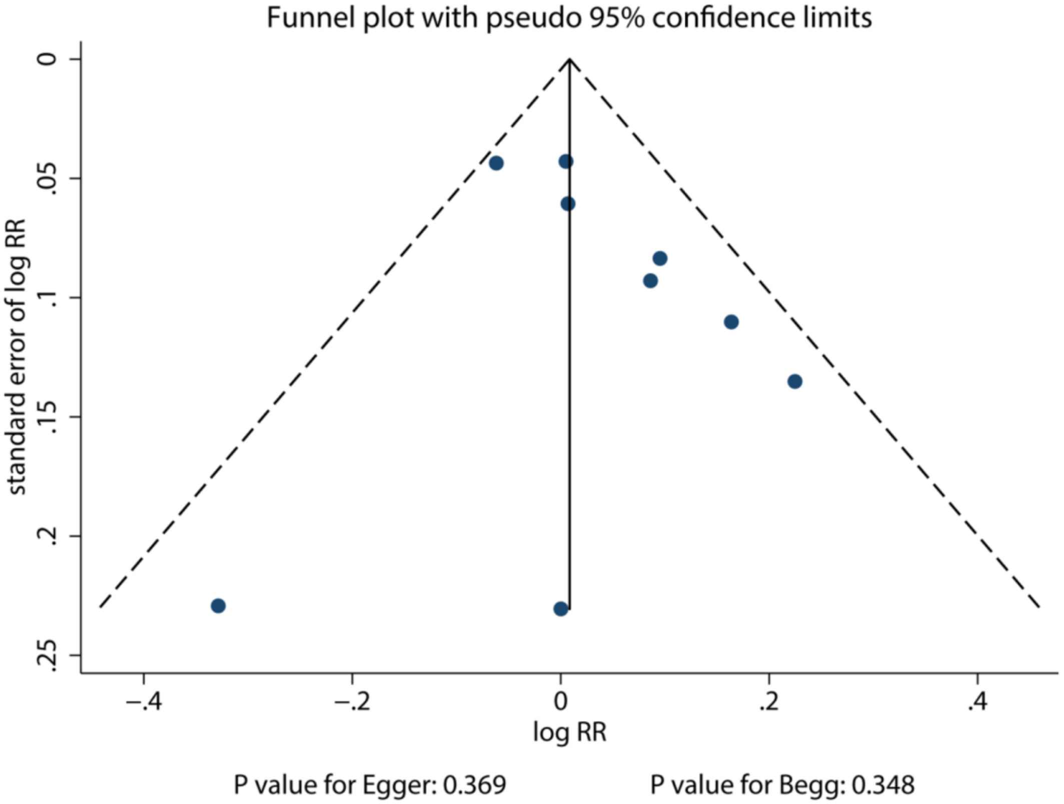

Supplementary Figure 31. Funnel plot for the association of subclinical hypothyroidism with the risk of any fracture.

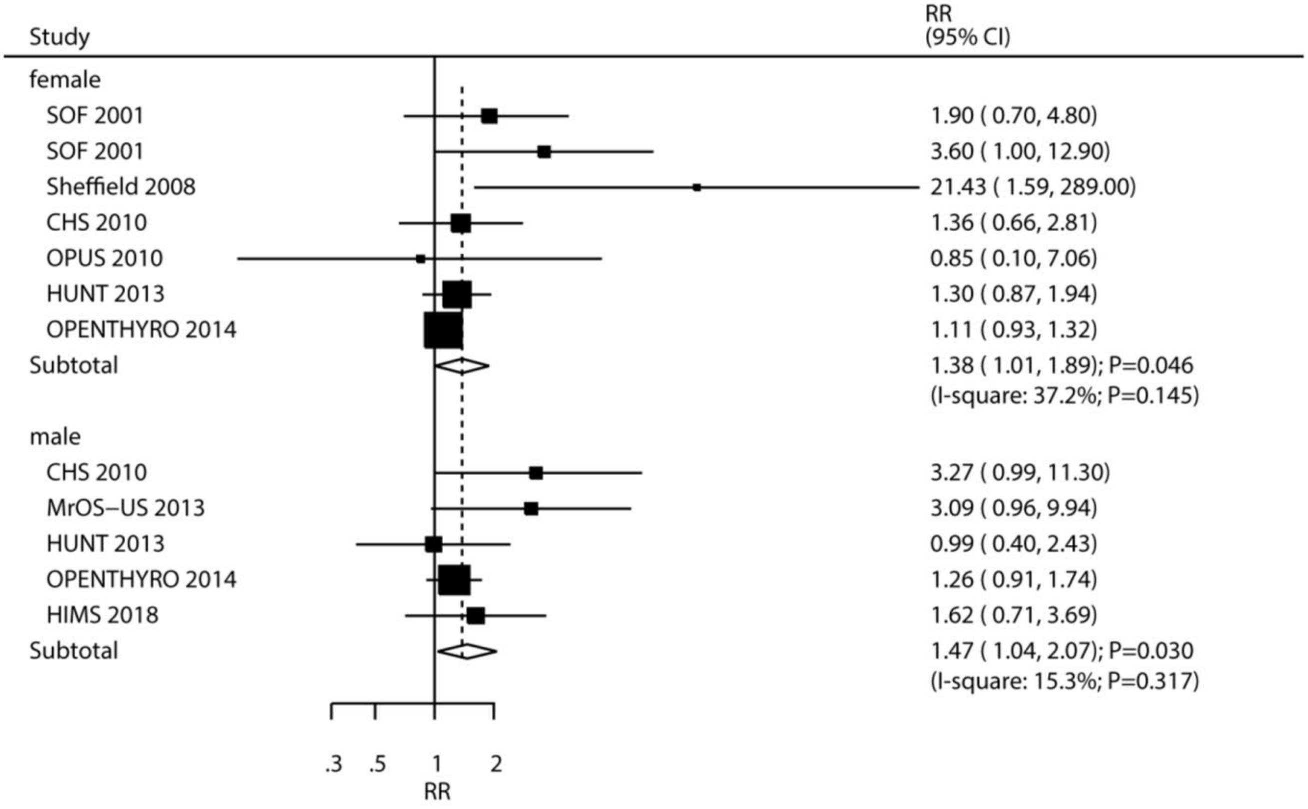

Supplementary Figure 32. Association of subclinical hyperthyroidism with the risk of hip fracture.

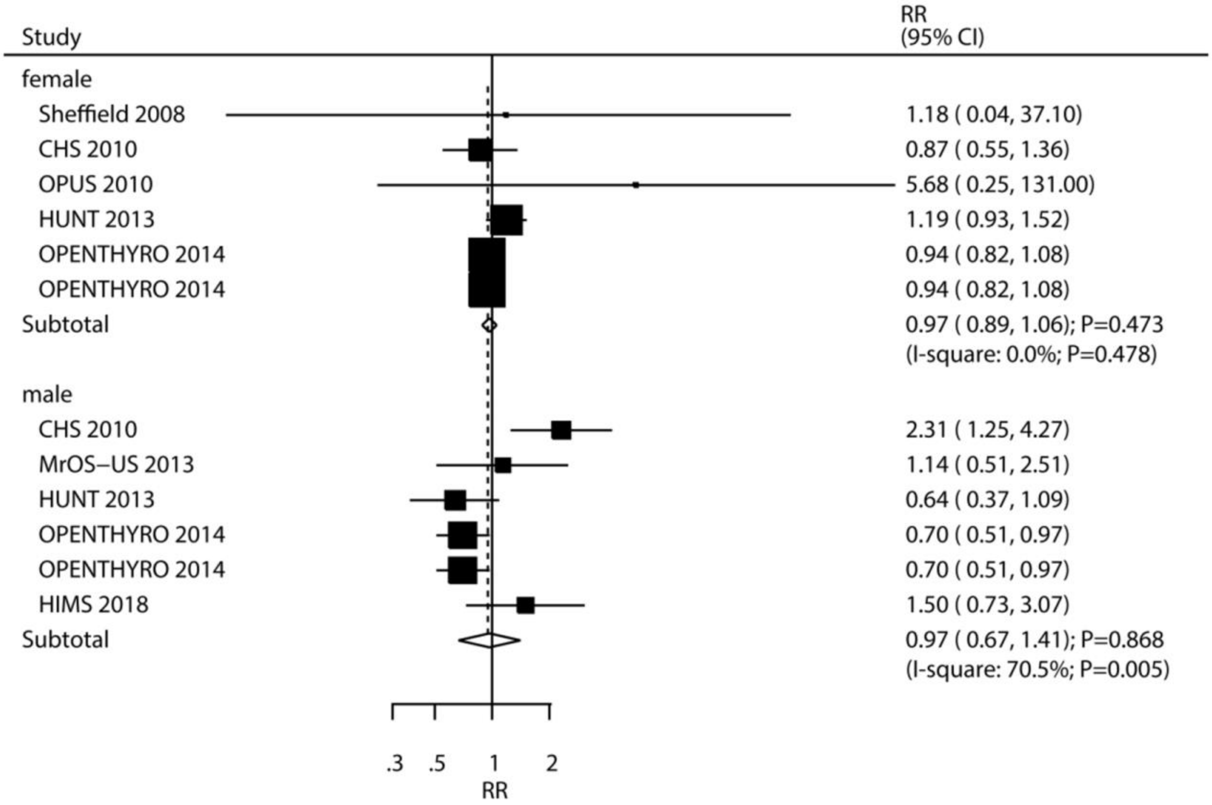

Supplementary Figure 33. Association of subclinical hypothyroidism with the risk of hip fracture.

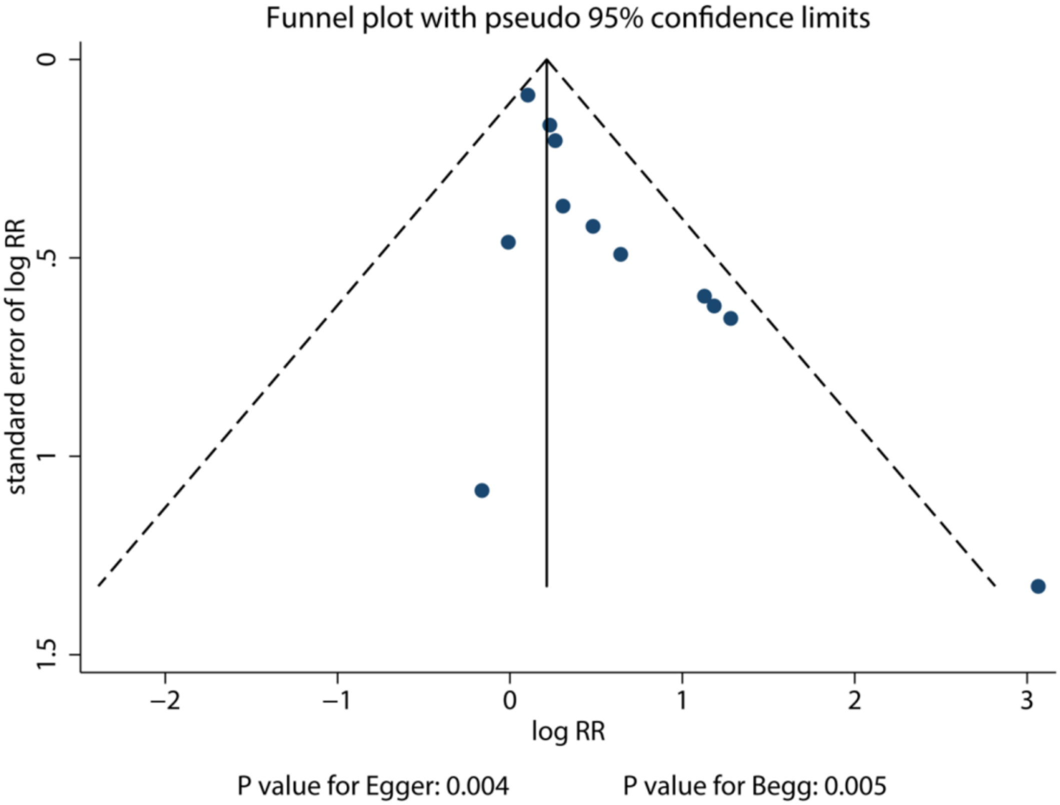

Supplementary Figure 34. Funnel plot for the association of subclinical hyperthyroidism with the risk of hip fracture.

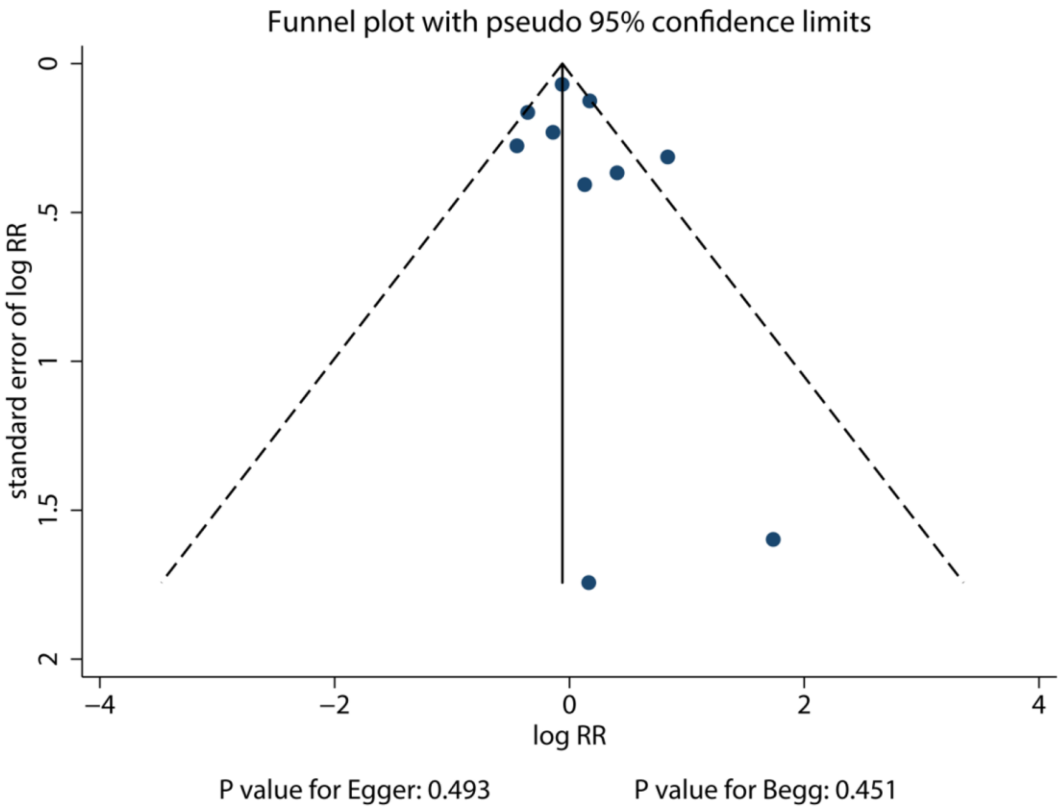

Supplementary Figure 35. Funnel plot for the association of subclinical hypothyroidism with the risk of hip fracture.

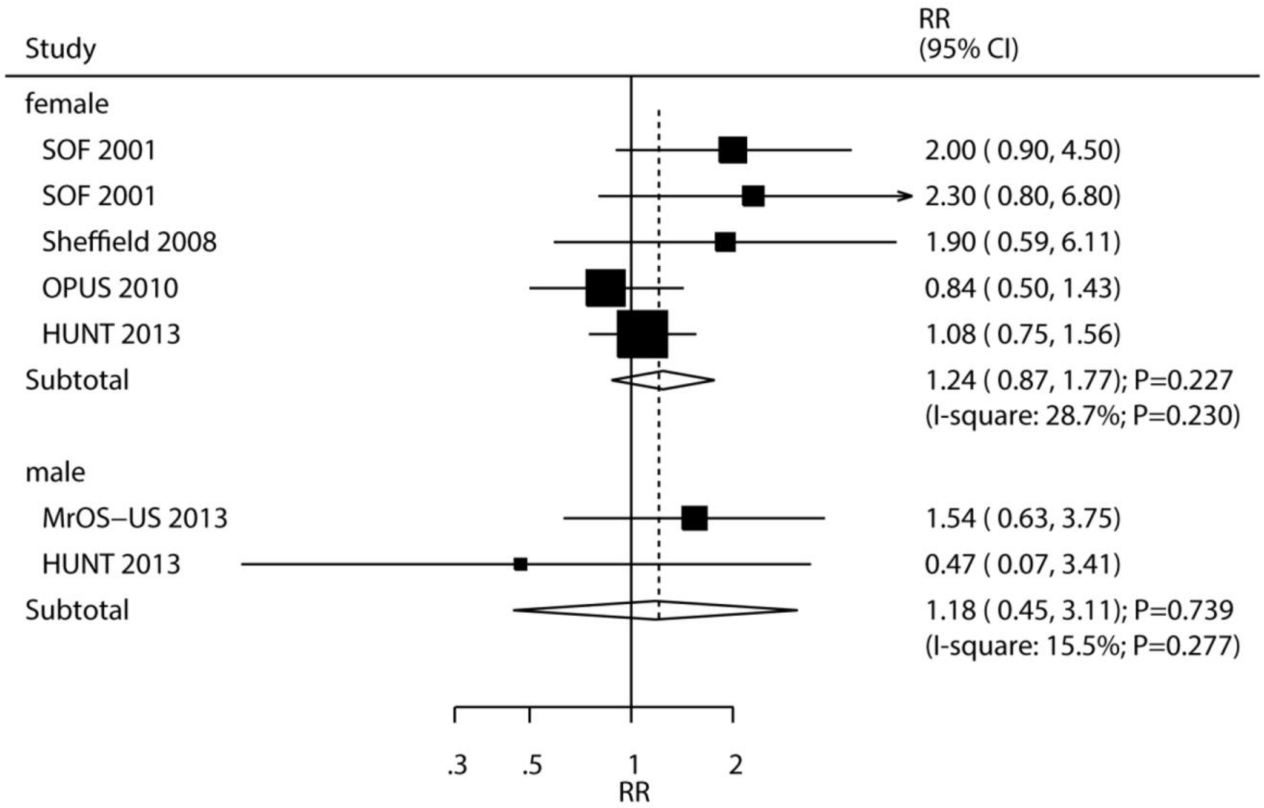

Supplementary Figure 36. Association of subclinical hyperthyroidism with the risk of non-vertebral fracture.

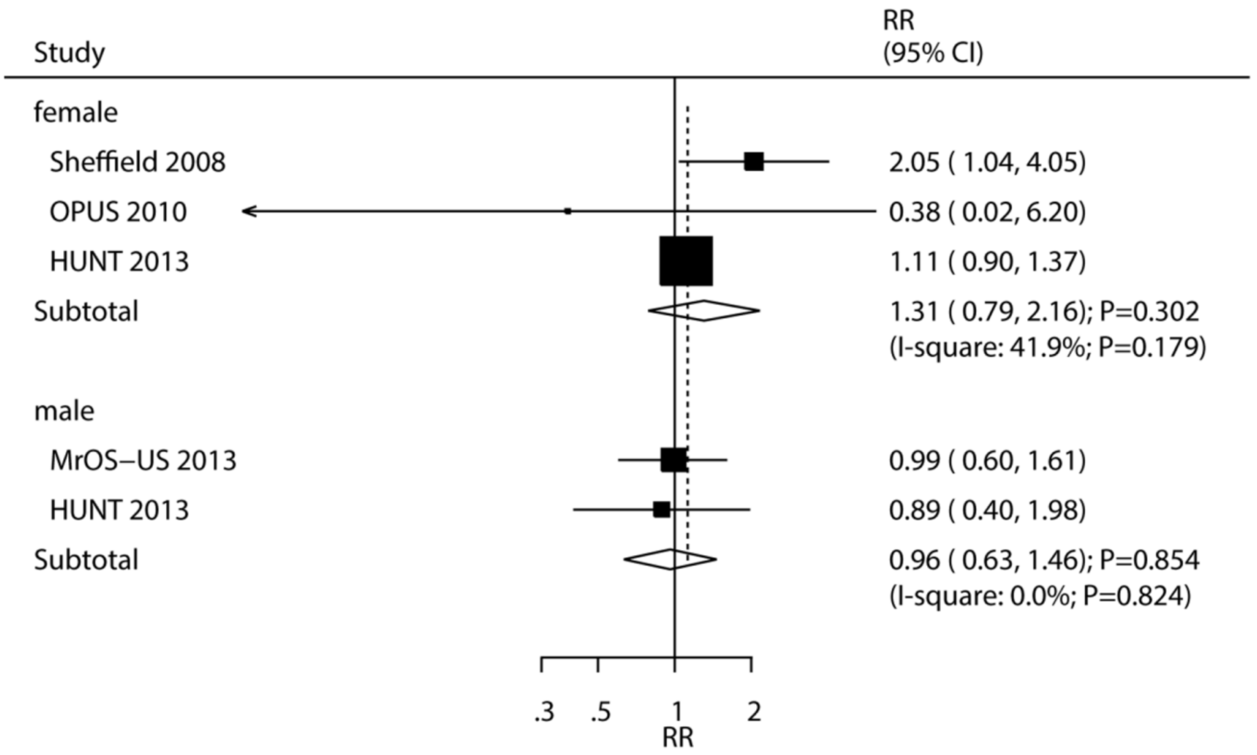

Supplementary Figure 37. Association of subclinical hypothyroidism with the risk of non-vertebral fracture.

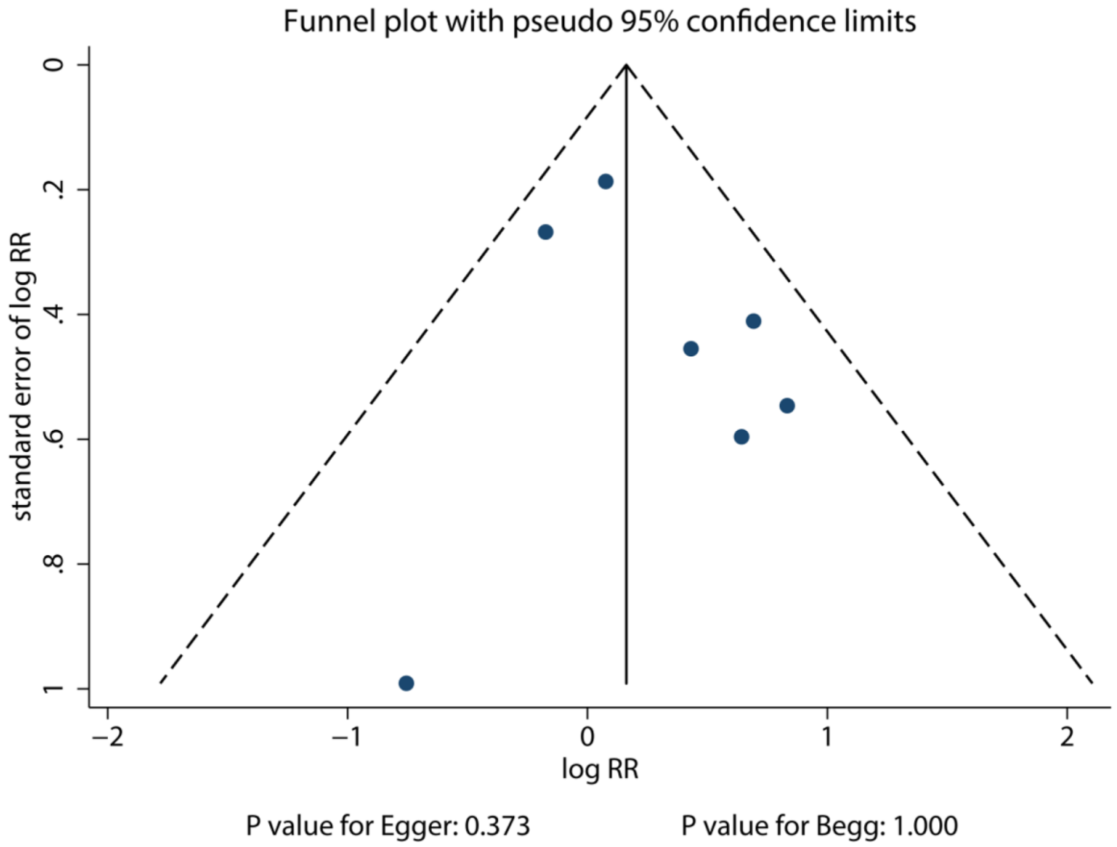

Supplementary Figure 38. Funnel plot for the association of subclinical hyperthyroidism with the risk of non-vertebral fracture.

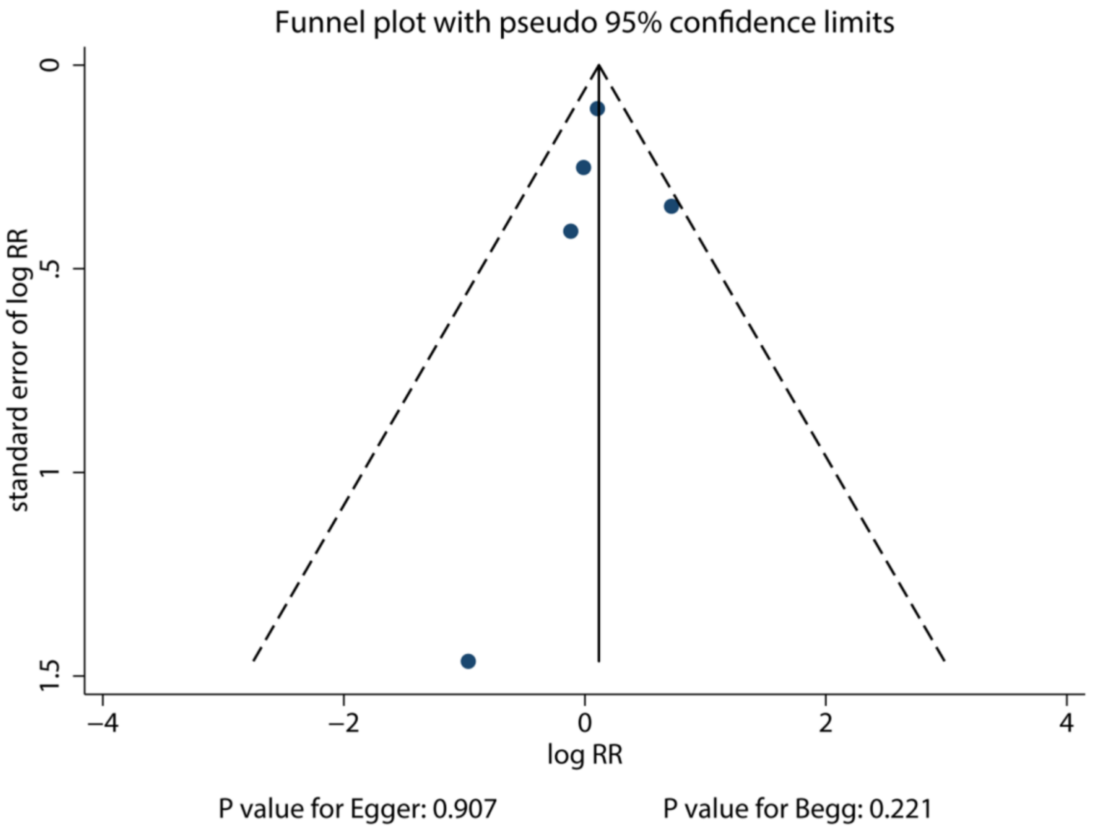

Supplementary Figure 39. Funnel plot for the association of subclinical hypothyroidism with the risk of non-vertebral fracture.

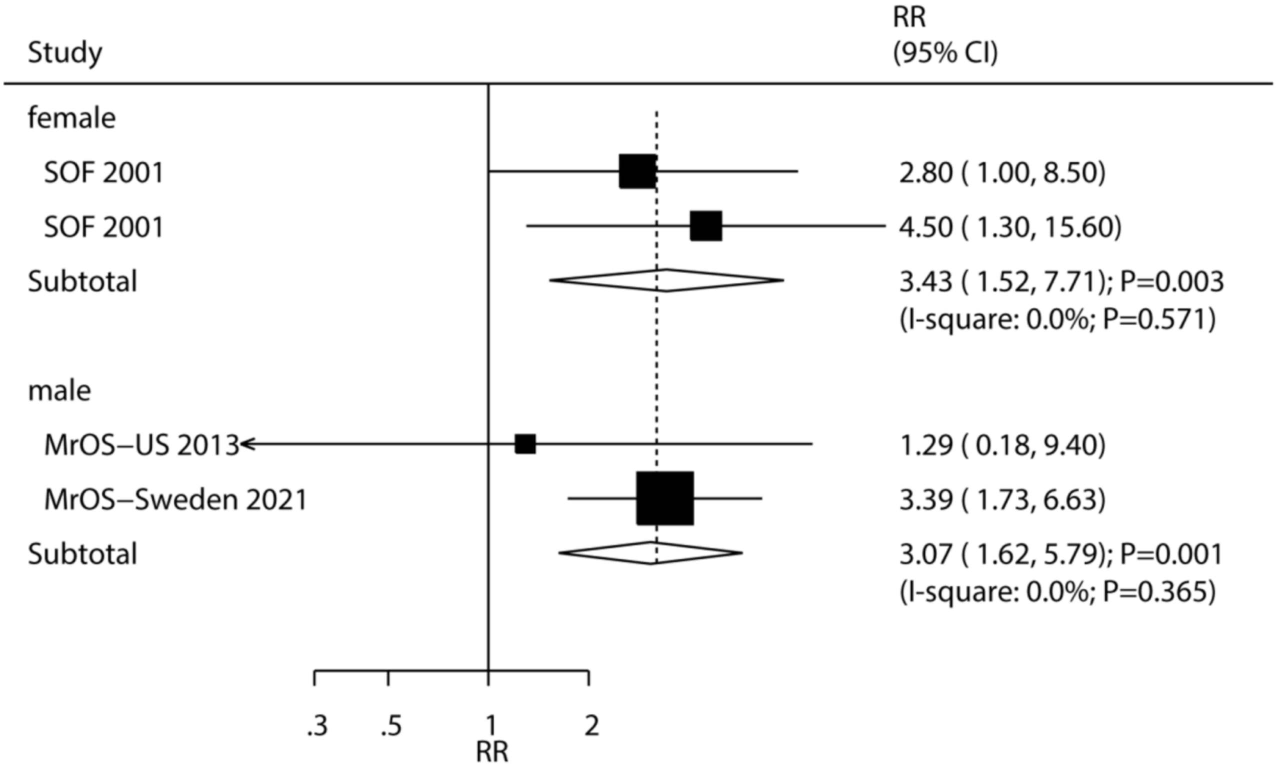

Supplementary Figure 40. Association of subclinical hyperthyroidism with the risk of vertebral fracture.

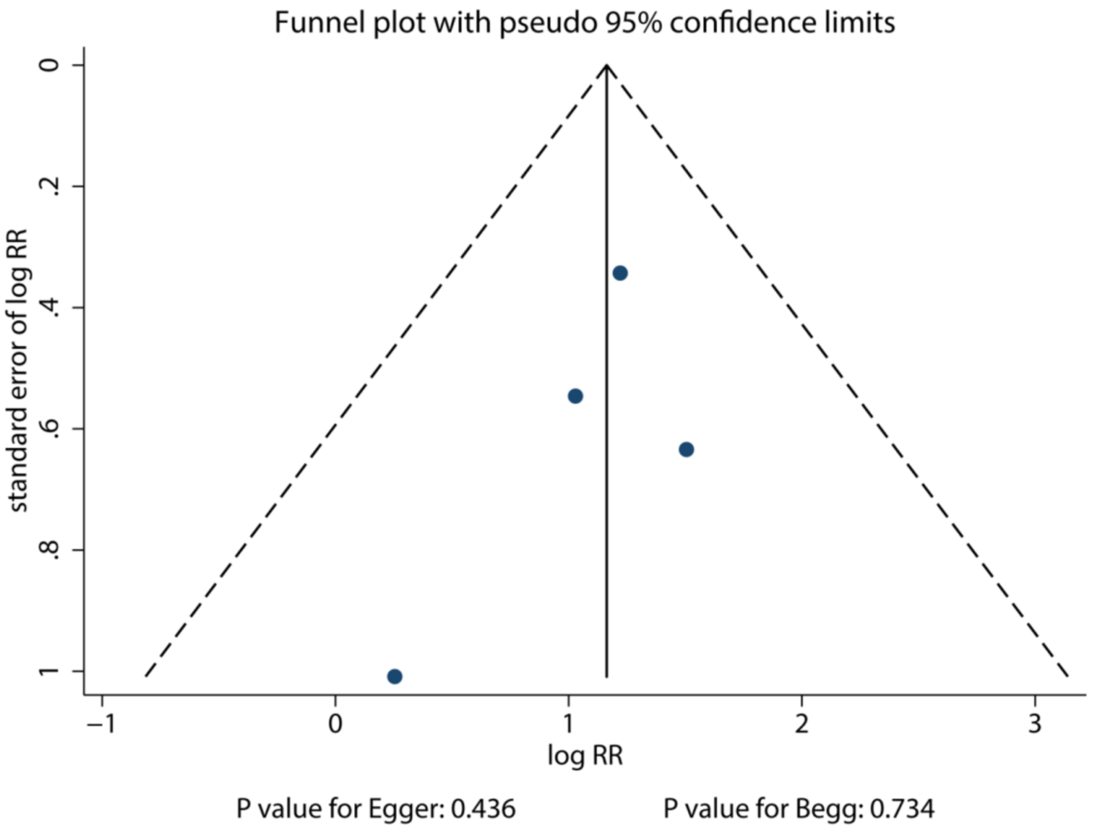

Supplementary Figure 41. Funnel plot for the association of subclinical hyperthyroidism with the risk of vertebral fracture.
