# Supplementary material for: Sex differences in major cardiovascular outcomes and fractures in patients with subclinical thyroid dysfunction: a systematic review and meta-analysis
Source: Aging (Albany NY). 2022 Oct 25;14(20):8448–85. doi: 10.18632/aging.204352 (PMC9648794; doi:10.18632/aging.204352)
Supplement: Supplementary Table 1 [file aging-14-204352-s002.pdf]

SUPPLEMENTARY TABLE

Supplementary Table 1. Quality scores of prospective cohort studies using Newcastle-Ottawa scale.

Study	Selection				Comparability	Outcome			NOS
	**Representativeness of the exposed cohort**	**Selection of the non exposed cohort**	**Ascertainment of subclinical thyroid dysfunction**	**Demonstration that outcomes was not present at start of study**	**Comparability on the basis of the design or analysis**	**Assessment of outcome**	**Adequate follow-up duration**	**Adequate follow-up rate**	**Overall score**
Rotterdam 2000 [16]	*	*	*	*	**	*	–	*	********
SOF 2001 [17]	*	*	*	*	**	*	–	*	********
Birmingham 2001 [18]	*	*	*	*	*	*	–	*	*******
RERF 2004 [19]	*	*	*	*	*	*	*	*	********
Sheffield 2008 [20]	-	*	*	*	**	*	*	*	********
CHS 2010 [21]	*	*	*	*	**	*	*	*	*********
OPUS 2010 [22]	*	*	*	*	*	*	-	*	*******
PROSPER 2012 [23]	*	*	*	*	**	*	-	*	********
DNPR 2012 [24]	*	*	*	*	*	*	-	*	*******
MrOS-US 2013 [25]	*	*	*	*	**	*	*	*	*********
HUNT 2013 [26]	*	*	*	*	*	*	*	*	********
WHI-OS 2013 [27]	*	*	*	*	**	*	-	*	********
OPENTHYRO 2014 [28]	*	*	*	*	**	*	-	*	********
Ansung cohort study 2014 [29]	*	*	*	*	*	*	*	*	********
General Hospital Vienna 2015 [30]	*	*	*	*	*	*	–	*	*******
LDO 2015 [31]	*	*	*	*	**	*	–	*	********
USRT 2017 [32]	*	*	*	*	**	*	*	*	*********
HIMS 2018 [33]	*	*	*	*	**	*	–	*	********
US veterans 2018 [34]	*	*	*	*	**	*	–	*	********
Taiwan NHI 2018 [35]	*	*	*	*	**	*	–	*	********
THIN 2019 [36]	*	*	*	*	*	*	–	*	*******
NHANES 2020 [37]	*	*	*	*	**	*	–	*	********
Korean NHI 2020 [38]	*	*	*	*	*	*	*	*	********
MrOS-Sweden 2021 [39]	*	*	*	*	**	*	–	*	********
